# Supplementary material for: Feasibility of a self-management intervention to improve mobility in the community after stroke (SIMS): A mixed-methods pilot study
Source: PLoS One. 2024 Aug 13;19(8):e0286611. doi: 10.1371/journal.pone.0286611 (PMC11321569; doi:10.1371/journal.pone.0286611)
Supplement: S1 File — (PDF) [file pone.0286611.s001.pdf]

McGowan transcribe + translate

McGowan Transcriptions Limited
Meads Business Centre, The Financial Management Centre, 19 Kingsmead
Farnborough, Hampshire, GU14 7SR United Kingdom
t: 0800 158 3747 e: enquiries@mcgowantranscriptions.co.uk
www.mcgowantranscriptions.co.uk

Focus group recording - Intervention group 2 (P1) SEPTEMBER 2022 LC - DEPTH - 32 MINS
[MALE RESPONDENT]
[Other comments:]

I2: P1, are you okay with the recording?

I'm recording it, P1.

Yes, okay.

Thank you. [Long pause] Do you want me to do the interview with P1? I like talking to P1!

I2: Oh yes, please. No worries!

Yes.

All right, P1…

I2: If I have any questions, I'll just interrupt.

All right, then. P1, thank you so much for your time, for participating in the research study and also for giving us your time to do this interview just to collect data about what we did well, what went wrong and what we could do better. Thank you for that. Then I just want to know certain things about the design of the study. We're going to talk about certain things about your personal experiences as well. But you can feel free to say, 'No, I don't want to answer this question' or, 'I'm tired' and you can…

No, I'm never tired; I'm full of life!

I know! Hopefully it'll take about 40 minutes to an hour; it shouldn't take too long. It's because only it's you; if there are a couple of people, they'll talk a lot more and they'll keep saying things. So I

don't think it'll take that long, so we won't use your data. Even the stuff that you say, we will not put your name to that. We will just say: Participant S said this. You will not be recognisable for any of the data that will be published or written up.

Yes.

So feel free to converse; it'll all be anonymous. Also remember we are just talking about your view so there are absolutely no right or wrong answers; we just want you to share your experience with us. Feel free to critique. [?Ahmed] will leave the room in a couple of - after some time because he has to go and do screening so if you want to thrash us, please thrash us! Please be critical, okay? Do you have any questions before we start?

No, I'm all right.

Transcript	Codes	Themes
**Just for the record, I don't think - because this is recorded, I don't think you need to state your name, but why did you agree to participate in this study, P1?**		
Because I want to improve. I want to get better and I want to - I'd like it all now. I would like to run before you can walk, but obviously I've realised that you can't run before you can walk. You've got to take your time and do your daily exercises and that's where it's helped me, to be honest. I've done them, plus the whiteboard and my exercises from my physio and <u>I've seen improvements. That's what it's all about; improving.</u> You're not	Hope of improvement let them join the programme<br><br>Improvements were seen	Motivation<br><br>Perceived benefits

going to do it within a week and think, oh, this is a waste of time. You've got to carry on and carry on doing it; you will see little improvements. It might be weekly or it might be monthly, 'Oh, I couldn't do that before!' But I've definitely improved; I feel brand new. I'm still broken a bit, lie my arm; still not doing it, but I know I've still got to participate and carry on doing my exercises and try and get my brain to do my other bits.	Patient's Knowledge helps recovery	facilitators of SM
**You are very self-motivated, P1, I have to say that! You've already touched on the next question but I am still going to ask the question: in general was the study useful to you?**		
Yes, it was very useful. It was a challenge. Some of the exercises I still can't do because, I don't know, the brain to the muscle, that bit. Like lifting my heel to my bum, I still can't do that - but I'm working on it - but others… I can do some of the others really good and where I was doing 20, I can do 40 or 50. <u>I've seen the difference, the strength in my leg; it's been brilliant, to be fair.</u>	Useful programme<br><br>Recovery can be a challenge<br><br>Improvements were seen	Participants' evaluation of the programme<br><br>Challenges

**Excellent! I'm just going to ask you about every element of the study just to see where we could improve. So you remember the first education session, like slides that Ahmed did with you about stroke and self-management and all of that? I don't know if you remember that because it was three months ago, but was that helpful for you?**<br><br>Yes, <u>everything has been helpful because it just is part of your journey and you improve</u>, and what I've found helpful as well is, I've always done little videos of my journey of where…<br><br>**Really?**<br><br>Yes, so what I plan to do is obviously when I do retire, whenever that is - because I could go at 55 but I'm probably going to hold on for a bit longer - I want to give back to the hospital and do a day at the hospital voluntary and go around and speak to people. I'll have videos still on my phone and I've got it under just the recovery page between me and my wife.	The programme was helpful/ SM is part of journey	Perceived benefits<br><br>Participants' evaluation of the programme/motivations

**That's a really good idea! Oh God! That'll be amazing to help other people. The information that we gave you regarding the study or the self-management programme or in our conversations, was that easy to understand? Was it simple enough?**		
Yes, it was all simple and some of the exercises, obviously, some people are at different stages and they can't do this, can't do that. I could get on the floor easy and do the bridges and everything. I enjoy doing that, but then some exercises I couldn't - but I know we're all different. There are different strokes, basically. People are affected differently but you give it a go, you try your best and you do it safely! You've got to do a little risk assessment on yourself. You've always got to think first, stop and think. What we use at work; before you do anything, if there's not a risk assessment you've got to do a stop and a think so you don't injure yourself or have a fall.	Simple information provided	Participants' evaluation of the programme
**That's very true, what you're saying; safety comes first. You**		

**know the way we structured it? As soon as you came into the study we did some education and then we did some goal-setting with you and then you joined us for exercise classes. Then you had the session with another expert coming and talking about his experience. What do you think about the structure? Was it helpful? Was there anything missing?**<br><br><u>Yes, I thought the structure was good</u>. Me from a personal point of view, it was every say two weeks, the Zoom meeting. I would've liked every - once a week, me personally, because I felt a togetherness and you're meeting with other people. You would give them tips: like the sit to stand one; move your affected leg, which I learned off my physio and I let you two know. So then that was good; you brought that into the thing so I felt that leg then, you're pushing more up off the affected leg and it gets stronger.<br><br>**It's about helping each other and helping yourself, isn't it?**<br><br>Yes.	Good structure<br><br>more group sessions (every week)	Participants' evaluation of the programme<br><br>Suggestions

**What about the goals we set you? We asked you for your goals and we wanted you to get involved. We planned the activities; we said you can do this, this, this in order to improve your walking recovery. How did you find that involvement?**<br><br>I found that very good. I pass my goals kind of thing. I've done 10,000 steps and I feel I could do that easy now daily, but I'm now without my stick now, which is brilliant. I do find if you haven't got your stick and you're out in public like in Stratford town centre, busy Jubilee, no one knows what you're going through, your personal journey. They can't see the inside, that you're a bit broken or you're a bit unsteady, but if you've got a stick, people move out of the way. I would probably advise just take the stick even if you don't use it; just for busy places but not… If you're walking in the park and it's free, you don't need…<br><br>**Just as a warning?**	Happy to achieve goals<br><br>Walking more steps without a stick	Perceived benefits

Yes, I think something like that, really.<br><br>**You know when we were asking you to set your own goals, tell us what you want to achieve? Did you find it a bit challenging? Did you find that, 'Oh, I can't think about that'? Some people do, but did you find that a bit challenging?**<br><br>I found it challenging but it's what you want to get to, where you want to be; those goals are set, you set those goals, you reach those goals, then you set some more goals. Obviously then if you get those goals, you reach some more. It would be good for me to drive again, to play golf; they're my next set of goals. I just need to work really more on my arm. My legs are not bad, I would say. I'm 70 per cent probably with my leg.<br><br>**I think you've come into the habit of setting goals for yourself once you…**<br><br>Yes.<br><br>**You started in…**	Desirability to continue	Perceived benefits

McGowan transcribe + translate

Always got to do that, always set goals and small ones first, then achievable ones. You can't set, 'I want to run a marathon' because that's miles away.<br><br>**Unrealistic.**<br><br>You've got to be realistic; realistic goals, little things like even opening the door; I tend to try and always use the affected hand so try and open the door that way. You've got to try things. Obviously do things safely, hold a cup of tea, you've got to use both hands because obviously that one, you don't want to injure yourself. I'd get…<br><br>**Yes, start with cold coffee!**<br><br>Yes, I generally do it with just water, to be honest, because it doesn't matter if you spill…<br><br>**Yes, or cold coffee, something cold.**<br><br>Yes.<br><br>**You know we had other people coming and speaking to you and you had P5, P7 and everybody in**		

**the group who supported you, and you supported the others? What did you think of the support from peers and experts like [?Bren], who's had a stroke for many years?**<br><br>Yes, I thought he was very, very good. The lady who spoke…<br><br>**Oh yes, Liz came and spoke to you.**<br><br>Yes, I thought inspirational, but that's what I want to do; I want to be that lady, do you know what I mean? I want to be an inspiration to others and when I get better, that's my journal and my story. I can share it with others, so look, you've got to carry on every day. It doesn't matter. You're going to have your good days and bad days, and your ups and downs. You've got to get negative thoughts out of your head. <u>You've got to be positive. You've got to get the mind right, the mindset; that's where it all starts</u>. You must sleep; sleep is the biggest one because I've found I've come on leaps and bounds now. I'm sleeping regular, I go to bed at a certain time. I'm drinking more water every day. You've got to change your habits, just a few of them to make it	Peer support and talks were very, very good and inspirational<br><br>Motivation is important	Participants' evaluation of the programme<br><br>facilitators of SM

better. You've got to have your break, you've got to have your dinner, you've got to do this and that's probably things where I was always rushing and no time to talk, and rushing round and thinking, work, work, work.<br>But now it's made me realise it's good… That wasn't good for your health so it's changed. It's given me a different look out on life, to be honest.<br><br>**Great, P1, you're inspiring us as well. Yes, that is really, really helpful and I need to go back to Liz and tell you [sic] how much she inspired you. Thank you for that. You know the group exercises? Would you recommend that for other - in the future would you recommend that for other people with stroke to get together and do exercises in groups?**<br><br>Yes, I found that good, doing that. It was good seeing others, even if they were struggling. You could help them, then, you were talking to them, help them or, 'Try this' and then talking to them at the end, if the conversation was good. You could		

give each other help. One lady hadn't heard of the FES, so I said, 'Look, it's been brilliant. You just use it 20 minutes of a morning, 20 minutes of a night and just to get the brain ticking and work the hand.'<br><br>**Yes, it's useful to support others, very good. You know during the exercises at home on your own? We left the exercise booklet and you had to do the exercises on your own. You had to say what was hard, what was not straightforward. You had to evaluate your own performance without our help, so did you find any challenges? How did you find that whole thing of doing it on your own and evaluating your own performance?**<br><br>Because I'm motivated, it was good for me. Right, get up, have my breakfast, do this, do the FES. Right, it's ten o'clock, I'm doing my exercises. That was it and I'd do it and sometimes if the weather was good I'd do it outside. There were a few, as I said, that I marked down; I found it hard, the heel to bum one and then the - I know it was step 3 or something	benefits of group exercises (good seeing others, even if they were struggling)<br><br>Motivation is important for SM	Perceived benefits

like that, doing the bicycle. But I can do the bicycle now, it's fantastic! I lie down and I can do that, but I think that's helped with: keep having a go and you will achieve. You've got to persist in doing things, but I've been going to the gym. I went from the sit-down bike to sitting down on the proper bike and doing it, and I've felt better and I've challenged myself. Try and do ten minutes - and now I'm up to doing 20 minutes. Right, it's time and on distance so far I can get… I got to six kilometres in 21 minutes.<br><br>**Excellent! Honestly, like I said before, you put us to shame! You put me to shame. I'm not doing any exercise at all. You know you spoke a lot about being motivated and doing things on your own? Is there anybody else or anything else that facilitated you to push yourself and continue doing this programme?**<br><br>I think what it is, it's all in the mind. You've got to be positive and you've got to - don't feel sorry for yourself. Go out there, you've got to get outside as well in the fresh air because that's what I found; it was good, instead of		Facilitators of SM

McGowan transcribe + translate

sitting in that house with the four walls, you've got to get out there for your own mental health. It's been a challenge. People have seen me; my exercise is walk round the block of my houses, the big circle. I'd be gone sometimes for a while because neighbours have come out talking to me and things like that, but it's good and you've got to have that positive mindset whereas before I was this, I was that. Now he said, 'You look fantastic!' But I'm still broken a bit - but I know I'm going to get there, if you know what I mean. I'm going to get this wrist, arm and hand going, so it all starts in the mind, definitely. You have a positive mindset. I know you can be negative and what have you. You've got to be a better version of what you were before, a better version of yourself. That's what I'd say.<br><br>**Thank you, P1. The other thing is, like you said, it's all from internal, intrinsic motivation rather than your wife telling you or your family telling you.**<br><br>Yes, it's good to have a partner or a family member who pushes you. My	Motivation is important for SM	Facilitators of SM

wife's gone back to work full time now, but she was off for a bit. The whiteboard of the exercises and that, and she was like, 'Look.' She'd come in from work when she'd been… 'Have you done them?' I said, 'Yes, I've done them.' I was doing little videos of me doing them and you can see on the videos of it where I've improved.<br><br>**Excellent! That's such a good idea to video yourself. The study itself, a couple of people like you went into the intervention group and you did a whole lot of things. They've got another couple of people who did not get the intervention; they only got the pedometer and we set the goals for them. They didn't get the exercises, working in groups and all of that. They had a little bit of education. So the design itself was to see whether the programme is useful with the group exercise sessions in it, so what do you think of the design of the study? Obviously for research purposes we had to do that, but what do you think about the design of the study?**	Family is important for SM	Facilitators of SM

I think the design of the study is, well, very well organised and it's right in what you've got to do because obviously my wife did a lot of research while I was in hospital into strokes and got books and got the bands and did this and did that. A lot of it is the exercises, like what you've got, the ones like standing on your toes, leaning forward is to get that muscle going and things like that. I found that very beneficial to help with the lifting of the toes and things like that. Yes, I think it's well organised. The design is well done and as I say, just a few critiques, like I taught you that one with the foot; the affected foot. Changed that one to that one.<br><br>**Yes, that was a very useful strategy; that was a really useful strategy, thank you, P1. Ahmed and Jessica came and did some assessments on you, like the walking speed, getting up and going and all of that. Did you find those assessments useful, the outcomes, whether they were relevant to you?**	The design is very well organised	Participants' evaluation of the programme

Yes, I found them useful. Obviously when I first tested, I had my machine on, the FES thing. When she came, I said, 'Look, I haven't got my machine on.' She said, 'Well, do you feel confident you can do it without?' I did it without the machine, so yes, I felt… I don't know whether I was quicker or slower, but I've got to have the confidence to do it. Obviously I've been advised to use the machine, still, for getting the brain, the pathways to the muscles so I want to carry on because - yes, I'm not the expert. I'm just the patient. I've got to listen to the experts.<br><br>**Soon you will become the expert! Already you are the expert; you are telling us a lot of stuff which we can tell the other patients, so you are the expert already! Just out of interest, did you discuss the study with other people? I know you discussed - or was it P5, P7 who discussed it with you when you came on board?**<br><br>Yes, P5, P7 had discussed it. He said, 'Hadn't you been asked to do this study?' I said, 'No.' I said, 'Well, let them know I'd be interested.	Useful assessments	Participants' evaluation of the programme

Anything to improve.' What I did find; it can be lonely and obviously if my daughter has gone to university and my wife is at work, you're lonely. Unless your mate phones you from work or something like that, it can be lonely so I've felt it good to participate and have a chat to people and all that. That's what I've found; it was good for the mental health as well, like you didn't feel you're on your own kind of thing then. That's what I thought was very good.<br><br>**That's really good to know. With regard to what the NHS was giving you, how did this programme fit in with what else you were doing with NHS and the private therapy? Was it supplementary? Did it add, did it clash?**<br><br>No, it was good because I could let you know what I was doing because I knew the week before my appointments and things like that. So I was always okay and the private physio was always on a Saturday. NHS was usually a Monday, Tuesday or Wednesday and if you were a Wednesday I'd be in touch with them,	Good for mental health	Perceived benefits

'Can I have that Tuesday' or something. So it's been…<br><br>**Yes, we need that flexibility.**<br><br>Yes, it's been flexible and plus I've been lucky because I've had people - because obviously you can't drive yet; I've had people who have picked me up and taken me, supported me like my brother. He took me to the Solihull Hospital all the time. Solihull Hospital, they've kept me on but they're going to see me in three months. I know I've got to try and get this arm a bit better and the wrist and the hand move, so I've got to really concentrate on that, I have. I tell you what I have done, because my private physio says, 'I think you're ready for Connect 4.' I thought, no chance! But I actually had - I beat the wife three times as well, so there's nothing wrong with the brain! I probably bored her with how long it took me because I'd pick it up and I'd probably drop it, then I'd pick up, but I'd pick up and hold and then let go into the slot.<br><br>**Such a good exercise, yes - and you enjoy it as well, don't you?**	Easy to manage with NHS appointments	Participants perspective

Yes.<br><br>**Would you be setting your own goals in the future, continuing to do at home?**<br>Yes, I definitely am. Once I've reached those goals, I want these goals until I get back to as normal as you can. I know I probably have a few weaknesses in the arm. I might not be able to do this or what have you, but as long as I can get out and get about, I definitely want to get back to as normal as you can. You might not be 100 per cent, but even at 90 per cent I'd be a better person in that and I seem to look at things differently now whereas I see someone on a walking stick coming to you, whatever; I always think, oh, I wonder what's happened to them and whatever. I look at signs; have they had a stroke, with the arm or anything? It's given me a different outlook on life and so you've got to be - I don't know, you've got to be grateful.<br><br>**That's a really deep word, profound word; being grateful for things in life. This is about**	Desirability to continue	Perceived benefits

**improvements for this programme. What aspect of the programme can we improve in the future, for the future for other patients?**<br><br>I think I'm okay because I'm all right on the computer, like, but I don't know. Probably when you go around, probably show them what to do because a few of them were struggling, weren't they, to get on the Zoom at first? I'd say that, because then you're not waiting for others ten minutes, 15 minutes. People can switch off. I wouldn't because I just want to get it done and I want to carry on and do it.<br><br>**Yes, that's really valid.**<br><br>The exercises were all good and definitely what you need to help with the walking and all that. Before, as I say I couldn't lift that arm up, and now I can lift it. I know I'm pulling it with that but there's no pain. Before, I couldn't even get that so all the stretches, I know we did it with a cushion - or I did it with my stick - I felt it's got better.<br><br>**Thank you, that's…**	Teaching of how to use Zoom	Suggestions

I've improved.<br><br>**Yes, that's a really valid point about teaching them to use the Zoom and practising it with them on Zoom before we bring them into the group. Actually Ahmed did a little bit of trial but obviously it didn't go very well with everybody.**<br><br>Yes.<br><br>**My one last question is: you know we talked about our role, like how we communicate with you, to fix your appointments, to bring you in to the study, whether we were picking up your calls if you wanted to contact us, whether we were picking up your emails and stuff like that. Do you have any comments on that?**<br>I thought it was all good. I've got his number in my phone; I could send him, if he's on WhatsApp, a couple of the videos of in the gym now. It looks fantastic. Although I'm still broken a little bit, I've still got work to do but things like that, you could probably use WhatsApp as well, but it's nothing… It's good communication,	Happy with exercises<br><br>Better function	Participants perspective<br><br>Perceived benefits

<u>emails, Zoom and text message and actually call, which I didn't realise</u>. I just thought it was a normal time and he phoned me, 'P1, where are you? Are you at home?' 'Oh! Sorry!'<br><br>**I hope you didn't find it intrusive?**<br><br>No.<br><br>**I hope you've been…**<br><br>It's all good and communication is good, and as I say, it can be lonely being the stroke survivor at home. You can be lonely so it's good, so that's what I found, like me myself, because - I don't know. I don't get many visitors - only because I said, 'You can't visit me until six o'clock until I've done my exercises and done my daily routine.'<br><br>**Amazing! Thank you, P1. I've run out of questions for you. I'm going to stop recording, but I really, really appreciate your contribution and your participation and how you supported the others in the study. You were such a valuable member of the group so thank you so much for everything.**	Happy with communication	

**[END OF TRANSCRIPT]**		Participants perspective

GMT20230117-110632_Recording JANUARY 2023 LC - TRIAD - 64 MINS
[Other comments: Average quality telephone recording, plus the general manner and strong accent in which one of the Respondents spoke, made some of their speech unclear. Some grammar and syntax was also incorrect. Speech has been transcribed as spoken so as to not lose any meaning. Transcript lightly edited to clear speech only.]

We are recording now, so we'll just…

R1: Got in; there!

Thank you all about this meeting and this focus session. Actually the purpose of doing this is just to share your thoughts about experiencing this walking programme, and how was it, how we can improve it and make it better. Please feel free to share whatever thought you have. There is no wrong or right answer to the question; we are asking just to facilitate the discussion. So, feel free to share your thoughts. We actually need to clearly record your permission for the recording. So, if you don't mind, we'll go one by one: just state your name and that you agree to the recording of this session. We'll start with Mr [?P2 0:01:02.9] here. Please.

R2: Good morning, my name is [?Om P2 0:01:07.3]. I'm here in the meeting so we can get something out of it.

Thank you so much. Christine?

R1: Good morning, my name is Christine. I'm quite happy with the recording, thank you.

Thank you so much. P4?

R3: Hello, my name is P4, and I consent to being recorded.

Thank you so much. Do you have any questions before we start?

R1: [?Sheba 0:01:43.3].

I2: Sorry! Do you all know each other? Have you all met in previous exercise sessions or anything?

R1: Yes, we have.

I2: So, you…

R2: We met once, yes; we had one Zoom meeting that we…

R1: Yes.

I2: So, you'll know each other just…

R2: We know each other's faces; no, we don't know them but we know by face.

R1: Yes.

I2: [Signal breaks up 0:02:08.7] themselves - just to make yourself comfortable in this group; we want it to be quite informal. You can be more chatty if you know the person, isn't it?

R2: Yes.

R1: Yes.

I2: So, is there anything you all want to say about yourself? I can start the conversation: I am flying to India, I am travelling tonight and I am like a headless chicken now [laughs]! I am like a headless chicken running around trying to finish as much as possible, so that's me today [laughs]. How are you today, Christine?

R1: [Laughs] I am not particularly busy today, but I'd like to come in your suitcase!

I2: I would love to take you - maybe not this time; maybe another time, okay?

R1: Okay [laughs].

I2: Not in my suitcase; I will take you on my next seat, yes?

R1: Yes, that'd be lovely [laughs]!

I2: Thank you, Christine. How has your day been, P4?

R3: Okay, yes. I haven't really done very much today, so after this meeting I'll start doing some work.

I2: Yes, P4 does a very exciting job.

R2: What is she doing? What do you do?

R3: Oh, I'm a model scout.

R2: A what?

I2: Model scout.

R2: Oh, model - what is that? I've never heard of that.

R3: Oh, I cast models for jobs and stuff.

R2: Oh.

R3: Like TV commercials that you see online.

R2: That's why we see all the beautiful ladies and muscle men! It was you!

I2: Because of P4 [laughs]! How has your day been, Mr P2?

R2: Yes, very nice to be in this, present and meeting in the flesh. It's different; we had never done this sort of thing before because I am not a Zoom fan, really. I don't know, I am not happy with it but…

I2: You can't really hug people on Zoom, can you?

R2: Yes.

I2: When Mr P2 goes, I'm going to give him a hug [laughs]. All right, thanks everyone. Ahmed?

Transcript	Codes
**We're going to start with our first question now about your motivation to join this programme, so: why did you agree to participate in this programme - so if we can start here?**<br><br>R2: Yes, I think I'm always happy to help if I can, doing on research - anything - to improve things. That's the reason I just agreed and said, well, you can… It's not personal; it's general, 'Can you help with the programme and get something out of it? We don't know what.' I'm getting something out of it now by just talking here. Normally I wouldn't have done this, but I'm quite happy to take part, but that's it, really.<br><br>**I2: Thank you, Mr P2.**<br><br>**Christine?**<br><br>R1: Well, it's the same: if you can do anything to help in the future, because we seem to have got… The three of us actually seem to have done very well through the programme. So, therefore in the future if other people can get the same - to the same levels as we have, I think that's marvellous.	**Reasons for joining (happy to help doing research to improve things)**

**That's good, and how was the programme? Hopefully it was useful. How do you feel? Was it useful for you?**<br><br>R1: Very, yes. Following on from the physiotherapy that I had via the hospital meant that I was able to walk better, yes.<br><br>**I2: P4, please can we ask you: why did you join us?**<br><br>R3: Oh sure. I don't really know; I just saw it and I thought, oh, had a stroke - might as well do it! Yes, I wasn't really getting very much therapy from hospital, so anything that could help me recover was a good shout.<br><br>**I2: Fair enough, yes.**<br><br>**How did you find this programme so far? Is it useful?**<br><br>R3: Yes. Every day I do what I can. I haven't made it yet [laughs]; maybe I'll walk extra tomorrow to try to make up.<br><br>**I2: Thank you. Mr P2?**<br><br>R2: Any way if we can help, you know? There's a reason I'm here as well, but you are helping us by going in the physio. It gives you more motivation to move, so otherwise if we're not getting this sort of thing we won't be doing anything; we'd be lazy at home watching TV and so on, boring. But taking part, at least we are learning and meeting other people, and I hope the programme will be successful for the future. We are all learning. Life is about learning. We are more focussing on a particular area - which is good; I think it's only good for the	**Improved walking beyond NHS care**<br><br>**Reasons for joining (not receiving enough therapy from hospital)**<br><br>**The programme motivates participants to move more**

future for people, the new generation and all this sort of thing, if you can help by that.<br><br>**So, now we'll move to actually evaluate and talk more specifically about different parts of the programme that we have delivered. Starting from the patient education session when we met actually online and we did explain how you can do training safely at home at the beginning after assessment: how was that education session?**<br><br>R2: It's the education, obviously; doing things - am I doing it right or wrong? Obviously we can do something which is not quite right, but you're keeping an eye or watching and we're on the right road. We can do most things, but doing it wrong or right is different [laughs]! Having somebody, a third eye watching you and saying, 'Look, it's not quite right. Can we do it this way' - which is a part of the programme. We can learn from each other.<br><br>**Yes, and when we did it for that presentation online…**<br><br>**I2: The first time when…**<br><br>**Yes, the first time. Was it very clear information? Did you find the information…**<br><br>**I2: How did you find that session?**<br><br>**…easy and clear?**<br><br>**I2: It's been a long time so…**	**Learning of how to do things correctly helps SM**

R2: On a long Zoom?<br><br>**Yes.**<br><br>R2: I'm not very good at - I'm a bit too lazy. I don't know about you, you know? The privacy but like I say, I will look into it more. My son is always on Zoom because he works, and my daughter is the same. They are on all the time but for us, the old people, we are trying to resist it - which we can't - but we try to. It's only because obviously it's nothing more than… Once you get to know how to operate it, we'll all be the same.<br><br>**I2: Yes, true.**	
R2: It's taking time to be open to that. That's my feeling anyway; I don't know if it's wrong or right, but I'm more of a private person, you know? How are you going to get help by sharing with other people, all the better.<br><br>**I2: Christine, you were laughing just a little while ago about Zoom and the education session. How did you find the education session that Ahmed did right at the beginning?**<br><br>R1: Well, yes, I enjoyed that too - but just the same: I am useless with Zoom. If my husband hadn't arrived back from the GP, I still wouldn't be with you [laughs]! I am not able to use Zoom at all so he's better at it than me, I'm sure.<br><br>**I2: Was the information and all that clear? Was it simple enough to understand?**	**Don't like online communication**<br><br>**Not able to use Zoom**

**When we talked about safety issues and how you make the training within a tolerable level and you do start with warming exercise and finish with cooling down.**	
R1: Yes. Also something that I found very, very useful was filling in the diary. Having that each day to complete, I found that really useful as well as the Zoom sessions.	**Recording diary and group sessions were very useful**
**We will come to that later.**	
R1: Oh sorry, am I jumping the gun [laughs]?	
**No, it's all right. It's really helpful, yes.**	
**I2: This is helpful, yes.**	
**We can ask that again.**	
**I2: P4, how did you find the patient education session, the first session?**	
R3: No, it was good. I was quite sporty when I was younger, so the warming up and cooling down I already knew but it was… No, it was good.	**Previous knowledge of exercises helps SM**
**I2: Do you think we can improve on that? Is there something you need more as information? That was plain information, wasn't it; Ahmed was talking at you, wasn't he?**	
R3: No, I found that fine, to be honest.	

**I2: Brilliant.**<br><br>R3: Yes.<br><br>**I2: Thank you so much.**<br><br>**Now we want to have your feedback about the programme. What do you think of the structure and content of the intervention, including everything: recording your steps and making goals and setting an action plan and everything? How was the structure and content of the programme?**	
R2: I think it was very good, in a way, you have a set programme to go by and trying to work as closely as you can - but human nature; we all try not to do it because it's so difficult physically. Some [unclear words 0:13:46.2] give you three or four [unclear words 0:13:50.1] and otherwise if you are not [unclear word 0:13:54.1] you are not... You say, 'Okay, I'll leave it to tomorrow, I'll do it later' or just lazy. As a human being we just try to get away with it if we can. That's being just...	**The content and structure of the programme was very good**<br>**Physical challenges related to stroke**
**I2: So, he was like the Big Brother watching?**<br><br>R2: Yes.<br><br>**Did you like that from us?**<br><br>R2: Well, I didn't say like but…<br><br>**I2: Bless Mr P2! He's being so honest, isn't he? Yes.**	**Researcher was like a big brother watching**

R1: He is.

R2: But you have to admit, life is - we've been brought up in such a way not to give your view, maybe wrong or right, but we're not machines or we all do the same thing. That is not life. We all find something very difficult and something very easy and we are, are that way. We resolve [unclear words 0:14:55.7] but it's pain; we were in pain after that, and already coming out is taking time. At least with the programme we're all getting - moving forward, not maybe as fast as we would like, but it's moving. We can see something, much of an improvement at the end; see a light at the end of the tunnel. That is my view anyway, yes. I am lazy, but I know at the back of my mind, only I can do it. If I can do it, anybody can do it as well but at the same time, it's a benefit for me, not anybody else. That's about me anyway. I'm not always active, or try to wiggle out of it.

We can all do that; it's always an easy way out. Just close your eyes and then - but you're not getting better, you're not improving. You are blaming somebody else. Go to the doctor; they've got to give me more medicine. It's not about that; it's working with whoever you're working with - doctor, specialist, people like you - trying to find out how they can help us. You'll push people further, I think. We are working, but what level [laughs]? Yes.

I2: True. How did you find the parts of the programme, Christine? Like you said the diary, the goal setting that Ahmed and I did, the Zoom sessions: every bit of the programme, how did you find that, Christine?

R1: I found all of it very useful because I started off just about being able to move with a frame and to walk, and now I can walk in the house just using a stick - which is brilliant really - within just a few months.<br><br>**I2: Excellent!**<br><br>R1: Yes.	**Very useful programme/Improvement in walking**
R2: It's only because of the programme, I think, we're doing this: it's only because of the programme, you pushing us. What she's saying, it's that a little bit of push you've given us.<br><br>R1: Yes.	**Improved because of the programme**
R2: That's what motivates; if we didn't do that we'd have thought, okay, I'm happy what I'm doing, you know? I think this is where your programme is quite handy not to limit what you can do. It motivates you.<br><br>R1: Yes, it gives you that extra push, doesn't it, I think.<br><br>R2: Yes. Life is about learning as well and at the same time, I don't think I would be in this position so when you have it, someone to help us, it's a great help itself.<br><br>R1: Yes.	**Motivated by the programme for better improvement**
R2: Not just going to the doctor once a week and giving you medicine and forget about it. Physio is something very new to me; I never even heard of that, but being through the	**The programme made people appreciate physiotherapy**

programme I'm really lucky to be in the programme as well at the same time.<br><br>**P4, what's your thought about the structure and content of the programme as a whole?**<br><br>R3: I did find that it gave me that extra motivation to exceed my previous scores - which is good - and then week by week I can see, track my improvement. Yes…<br><br>**I2: Yes, go on?**<br><br>R3: Oh no, what were you going to say?<br><br>**I2: Yes, I said: is there anything that you found difficult or your needed to change within the different parts of the programme, filling up the diary or coming on Zoom sessions? Anything you want us to change or anything that can be improved?**<br><br>R3: When I get up in the morning, the first thing I do is to check my emails. I sometimes have to remind myself to go and fill the diary in, but that is just… I just have to set a reminder on my phone, that's all, to do that.<br><br>**I2: What about you, Christine; was there anything that you think would've been better done differently?**<br><br>R1: No, I don't think so. No, I found some of the exercises quite challenging. I obviously am older than the other participants and so I did find I couldn't always manage all of - the whole lot of them in one day. Generally I was okay with them, but just	**Motivated by the programme for better improvement/ can see the improvements**

sometimes I had to limit the amount of each exercise that I did. If I found I was getting too tired then I just limited the amount - but in the main, I managed to get through them all, which I was very pleased with because it's that extra push again, isn't it, to try and motivate yourself into doing a bit more. Just one or two of them I did find a bit challenging. Yes, my balance isn't very brilliant. I still haven't got good balance - but just continuing to exercise, hopefully that'll get better.<br><br>**I2: Definitely, yes.**<br><br>**Can we talk about the goal setting that we did, when we sat down and set goals for three months for your programme? So, how did you find that, and were there any difficulties in doing that? What did you like about it? Can you tell us about your experience with setting goals?**<br><br>**I2: Do you remember? We discussed what we want you to…**<br><br>R1: Yes.<br><br>**I2: …achieve three months down the line. Yes, we were planning for your future, weren't we?**	**Exercises were manageable**
R1: That's right, yes. I fortunately have managed to… As I had my assessment the other day, I was able to… I have, yes, achieved pretty well the goals that I wanted to, which…<br><br>**I2: Well done, you!**<br><br>R1: Yes.	**Goals achieved**

**I2: So, did you find the goal setting useful?**	
R1: Yes, very useful, because at the beginning you don't… You hope that you will achieve your goals, but you're absolutely not sure. As I say, I was walking with a frame, not very well. Now I'm walking quite well with a stick, so no complaints here! Really, really helped me, thank you, yes.	**Goal setting was very useful**
**I2: Brilliant! What about you, P4, about the goal setting?**	
R3: It really was helpful. I do tend to push myself to the point of - I think because I try to get through it more than anything else, but that's just me. That's just trying to build up my stamina to get to the next stage. Then sometimes I do find that I'm knackered the following day - but that's okay.	
**I2: Yes, so you're setting goals for yourself now?**	
R3: Yes. There's one thing I haven't fully managed to do, and that's to carry shopping. I'm still, with additional weight, my balance is a little bit off. That's funny because I have to carry a backpack to go to work and it's quite heavy, but any time I put it on, I try walking in a straight line. I try to walk on the - you know the corner paving stones on the road?	
**I2: Yes.**	
R3: Can't do that. Failed miserably [laughs]!	
**I2: Oh, maybe we should design some exercises to improve that aspect. When you reset her goals or you're doing group**	

**exercises, ask her to wear a backpack and do the walking back exercise.**<br><br>**Sure, I understand.**<br><br>**I2: How did you find the goal-setting session?**<br><br>R2: Yes, I think it was very helpful. I think your mind is more - not willing - but you shouldn't have to push, push. At certain times I found it easy, it wasn't difficult, but the brain is not active enough and sending the signal. But on the whole, the achievement was good. At least we can walk. After they tell you how to walk with sticks or help, but over the time period of the programme, you have an independence to go even to the toilet or just walk around with this. I think I wouldn't have done it, but in the end with this programme we are achieving, and we are moving forward.<br><br>**I2: Do you still set goals for yourself, 'I want to be able to get this done, I want to achieve this'? Do you still set goals?**<br><br>R2: Oh yes, all the time. I always make an effort to go. At the moment I am not driving, so with the physio I was able to go on the bus.<br><br>**I2: Oh, very good!**<br><br>R2: I've never been on a bus before.<br><br>**I2: Excellent.**	**The programme helps independence**

R2: Yes, the first time it's difficult - but as you go along, it's really helped me to get my independence because I went to the physio. She took me the first time and then I think that was the best thing for me to not just sit at home. I run my own business, it used to be, and all that; I've always been active. I am not a guy who sits in the chair. I'm more motivated, I move around. It would not just, I don't know [?all moving around 0:26:24.1]. I want to try to get back. Going on the bus is a first step.<br><br>**I2: Exactly.**<br><br>R2: I want to be independent and go to [unclear phrase 0:26:38.6] just to I'm lucky because I've got a strong family base and they do most of the things for me, but I don't want them to do that for me; I want to do it myself because I'm independent. I want to still be independent and that is my goal.<br><br>**So, this is really a good motivation for you to keep doing this?**<br><br>R2: Yes.<br><br>**We want to talk about your reflection when you attended that online session with the judge; I think her name Liz?**<br><br>**I2: Liz came and spoke.**<br><br>**Yes.**<br><br>**I2: I think P3missed it, didn't she?**<br><br>**P4 was there; you were there, right?**	**Motivation for independence**

**I2: Yes.**<br><br>**Yes.**<br><br>**I2: How did you find Liz's talk, P4?**<br><br>R3: It was really helpful, yes. It was really inspiring as well. More of that, please [laughs]!<br><br>**What do you think of involving or having some guests like her?**<br><br>R3: Yes, some more would be very helpful.<br><br>**That's good.**<br><br>**I2: Was he in that session?**<br><br>**I don't think so, no.**<br><br>**I2: No, Mr P2 was not there. Yes, I think…**<br><br>**So, we had somebody else; was it Kirsten or was there someone else?**<br><br>**I2: No, I think it was just us, P4.**<br><br>**All right.**<br><br>**I2: We're just asking P4 about a session where we had somebody with a stroke a couple of years ago. She came and**	**Inspired by senior patient's talk**

**spoke to us about her life, how she recovered from the stroke. She was the youngest judge in the country; she was appointed a judge when she was 39 years old. She had overcome - yes, amazing lady! She's so positive and she'd overcome abuse, a bad marriage she had survived, and a very bad stroke. The doctors had told her that she's going to die, and then she came out of it. So, like P4 said, it was very inspiring.**<br><br>R1: Yes.<br><br>**I2: If I can chop of all the personal elements and I can send the sound file, I'll send it to you and you can listen to it, because she is very inspiring. She's very, very inspiring.**<br><br>R1: That's it.<br><br>**I2: Yes, I'll do that - but P4, you found it useful, did you?**<br><br>R3: Yes.<br><br>**I2: Thank you, P4.**<br><br>**So, we want to ask you about: how do you feel sitting at home and recording your diary every day without having a therapist watching you or helping you with that? How was it?**<br><br>R3: For me?<br><br>**P4, yes.**	

R3: Okay, there are some days that I do forget, I will admit it. I'm like, 'Oh crap! Got to do that.' Yes, it's okay. I think for me, because I'm always on emails and writing and stuff, sometimes it just goes out of my head and I have to remember the day or two after and I'm like, 'Okay, how did I feel? Let's get this done.' Oh sorry…<br><br>**No, go ahead. Is it…**<br><br>R3: No, what were you going to say?<br><br>**Yes, so I was just saying: because our programme is built on self-management and to keep you for a while away from a therapist watching you all the time. So, we wanted you to get that confidence in just working on yourself and taking care of your exercise and training at home. Watching your safety and your training programme and all these things.**<br><br>R3: Yes, I'm going to get better at it. I think because just generally I get so many emails and stuff, I wake up in the mornings and I'm like, 'Right, I've got to sort out work' and then I get distracted so I have to try and… I have to physically make the time to make sure that I put it in the diary.<br><br>**I2: [?Got to protect 0:30:43.2] your health.**<br><br>R3: Yes.<br><br>**I2: What did you think about filling in your diary, Christine?**	**Recording diary might be missed out**

R1: I found it very useful, yes. I like to do the exercises and fill in the diary each day and make a note of the steps in the evening that I'd achieved. Some days I achieved more than other steps - more steps than others - but I found it very, very useful as I've said earlier [laughs]. I jumped the gun!<br><br>**I2: No, that's very good. That just shows that they're very useful; that's why you wanted to tell us straightaway.**<br><br>R1: Yes, I found it very good. Yes, thank you.<br><br>**I2: Thank you. Christine. How did you find it, then?**<br><br>R2: Yes, I find it okay - but I'm not a diary man, you know? I'm more physical, doing things. Keeping a diary obviously is good, but I'm afraid I'm not very good at that.<br><br>**I2: That's all right [laughs]!**<br><br>R2: Yes, it was good - nice to have a diary; that's the programme you follow, you know? I believe in all that, but actually doing it is something different than… If you find it very easy, maybe you keep a diary, and I am a person who likes to have not too much of a diary.<br><br>R1: Yes.<br><br>**I2: Yes, keep it all here.**<br><br>R2: Yes, at the same time there is something more can be done around actually on the brain side of it, you know? Something can be improved that your mind is active. I think doing the	**Self-monitoring and recording diary was very very useful**

puzzles or something like that, I think this way they can improve, yes. Just doing the puzzles also, it was activating your mind. If your mind is active then [unclear word 0:32:54.6] your body. That's what I believe in anyway.<br><br>**I2: Yes, that's true.**<br><br>R2: Your body is then trying to act. If your mind is wandering or not doing - your mind is somewhere else and you're still doing the exercise, you're not getting 100 per cent out of it. I think some sort of programme where your mind can be more exercised.<br><br>**Yes.**<br><br>**I2: Are you suggesting these kinds of self-management programmes should have something to do with brain activities as well?**<br><br>R2: Yes, that's what I'm saying.<br><br>**I2: Brilliant, that's helpful.**<br><br>R2: Your mind is a… If your brain is - yes, I find sometimes if your brain is not there, for one reason or another, I can't explain, looking to [unclear words 0:33:42.6] that can be improved. This is where the programme can…<br><br>**I2: Excellent, yes.**<br><br>**Part of the evaluation of the programme, we want to see what helped you to carry out the programme to your best**	**Addition of Brain activities is suggested**

**at home. So, if we can ask everyone: what do you think helped you to do the best of the programme to your ability at home? P4?**<br><br>R3: Oh, I found the Zoom things really helpful.<br><br>[Over speaking 0:34:19.6]<br><br>R3: Yes.<br><br>**The zoom thing. Okay, great.**<br><br>**I2: So, was there anybody… Sorry P4. Was there anybody who supported you, encouraged you? Or was there something, some aspect in your life that motivated you to do the programme well?**<br><br>R3: I just want to get back to being healthy, just being able to do things for myself.<br><br>**I2: So, it's mostly your self-motivation then?**<br><br>R3: Yes. It's because I'm not driving yet as well so most of the places - I either have to rely on my mum to take me or I have to walk or get the bus. Getting the bus is also very fun and trying to navigate when the bus is moving and when it's stopping, and carrying additional weight on my back because my balance is kind of off. So, that is always a good time to try to do - but yes, I do find that I've been playing more online games just to keep my brain active as well. So, anything that could be interacting - what's the word?	**Self-motivation for improvement**

**Interactive?**<br><br>R3: Yes. Into the programme, that would be really helpful.<br><br>**I2: Christine, what about you: what has been motivating you, encouraging you, supporting you with the programme?**<br><br>**At home.**<br><br>R1: My family, really; they like to know how I'm doing all the time so they help me with… They've helped watch me with the programme, and grandchildren have helped to… Yes, they've kept [signal breaks up 0:36:10.5] making sure I'm [signal breaks up 0:36:12.7]…<br><br>**That was really obvious with her participation: sometimes I got late in giving a notice for next week's programme so I got contact from her daughter or daughter-in-law just asking me, 'Ahmed, when is going to be the meeting next week or what are we doing next week?' So, they reminded me every time before I contact you.**<br><br>**I2: That's so lovely!**<br><br>**This is very [?helping 0:36:37.8], yes.**<br><br>R1: Yes, very supportive.<br><br>**I2: Brilliant. What about you, Mr P2, at home or in your community; what supported you to engage in this programme?**	**Family involvement is very helpful in the programme**

R2: Well, family, you know? My son, they were all helpful by doing things for me - and it's no trouble - but that's probably why I found it so easy. The help is there and they… <br>**I2: Did they say, 'Oh, you have to get on and do things with the programme'?** <br>R2: Yes, all the time. <br>**I2: All the time, okay [laughs]!** <br>R2: 'Have you done this, have you done that?' I found it sometimes a bit frustrating, yes. <br>**I2: Thank you.** <br>**So, we're going to move to other part of the study. We have finished talking about the programme and content of the programme. Now we want to talk about the methodology and the design that we have used. As you might know from the information sheet we shared at the beginning of the study with everyone, we had two groups in the study. One of them was your group - which is the intervention group where we delivered the self-management programme for three months - and the other group who didn't; who only had the education session. This is part of the process actually to evaluate how the programme is effective or not. So, what do you think of the design that we have used for the study? P4?**	

R3: I think that the design, it has been - no, it has been really, really helpful because I didn't really get very much from the hospital, coming out. They had a few physio sessions but that was it. It was just like, 'Okay, you're on your own.' So, having that additional help and the design of the programme, giving me more structure in how to do more things, was really, really helpful for me.<br><br>**I2: Brilliant. If you had gone into the group that didn't get the intervention, like the whole programme, would you have liked it? Would you have preferred it or was that…**<br><br>R3: I probably wouldn't have done as much; I probably need to be a bit pushed to do more.<br><br>**I2: That's good to know. What about you, Christine? You know there was a group of people who didn't get any of this intervention that the three of you got, so what do you…**<br><br>R1: No, that wouldn't have suited me at all. No, I'm much happier the way - this way; the way the programme has been for us, yes.<br><br>**Yes, I just want to say: if you were lucky that you're assigned to this group, actually we didn't have any influence or any selection of people to assign to any certain group. We only used a computer-based programme which just throws people in this on their ID number as a participant and puts them into one of the two groups. So, just to be fair, we didn't choose - but this is part of the process of any intervention; to have two groups to see if the programme would make any change in their conditions or not.**	**The design was really helpful**<br><br>**Liked the push by study team**

**I2: Yes, it's just the way the research works, but we get your point from what P4 and P3are saying. We get your point that people don't prefer… If they are going into an interventional programme they don't prefer to go into a control group. Why would you join a research? That is my understanding from what you are saying; that you benefit from the intervention, so maybe that is not the best design for a rehab intervention study. Maybe that's something that we need to think about. What did you think, Mr P2, about having two groups where one group got the intervention and one group didn't get the intervention?**<br><br>R2: I'm obviously very lucky to be in this group and be active, but as you say, you didn't have a choice, we didn't have a choice. At the same time, you have to - either you're in or out. Some say, 'Well, I don't want to be in this study.' For that individual it works both ways: we are lucky we are active and more active than just… Obviously anything can be improved - that is life; you're always improving [laughs]. That is why we're in everything bad.<br><br>**I2: Yes [laughs]!**<br><br>R2: If you stand still we'll [unclear words 0:41:48.8] so that is my belief anyway. I still think the brain is the main area that needs to be looked at.<br><br>**I2: Focussed more on, yes.**	

**We had some people from the other group who didn't receive any intervention - but as you say, they had active brains and they were working on themselves.**<br><br>R2: Yes.<br><br>**They had the improved a lot.**<br><br>R2: Oh yes?<br><br>R1: Oh!<br><br>**Yes, they were for another group because they didn't wait for anyone to help them; they were seeking help for themselves, looking for things online, going to other support groups and doing maybe daily training with them in gym or in community centres. So, there are a lot of opportunities, especially after COVID. There have been a lot of programmes that started in the community just to cover the shortage in NHS community services.**<br><br>R1: Oh, that's good!<br><br>**Yes, so we'll move to the next question about outcomes, so if you remember the assessment that we have done every time we visit you - or doing an online assessment for P4 - we were testing how distance and how good you can move. Also there was another assessment for your general health and your quality of life in general, including walking and other activities, and also another test for your cognitive status and for depression. So, what do you think of these outcomes? Do you think we can add more outcomes? Or these**	

**outcomes are enough, like do they reflect what you are looking for from your rehabilitation programmes?**<br><br>**I2: P4, do you want to go first?**<br><br>R3: Yes, sure. I think it was harder for me doing everything online just because you couldn't fully assess how I was walking. I think the more that I… My walking is fine; the more that I exercise, the more you can see the disparity in the walking, so I think that kind of made it difficult for me.<br><br>**Yes, so this is one of the things.**<br><br>**I2: Yes, what about you, Christine; how did you find the assessments?**<br><br>R1: Very good, thank you. Yes, I was very tired after [laughs]!<br><br>**I2: Sorry you got tired, Christine, so sorry - but was it useful? Can it be improved?**<br><br>**Did it cover aspects of life that you want to improve after the stroke?**<br><br>R1: Yes, quite a lot of things I'd like to improve - but they don't seem to be happening [laughs]! No, but that isn't anything to do with the programme. I have managed the programme, but I'm still unable to walk unaided outside. I have a family member always with me. I haven't been anything - I go in cars, but that is only when I've been helped to get to the car, in and out. My body just doesn't let me, but as I say, I've improved so much. I can walk around the house and I can - my daughter's got me a	**Online assessment was harder**

wheelchair now so that… I was able to walk round one store but that was all, so she'll now take me shopping in my wheelchair. We can go to a number of stores - which is very helpful for me - but hopefully I'll eventually be able to walk around a couple with just my stick or my walking frame.<br><br>**I2: Excellent. How did you find the assessments, Mr P2?**<br><br>R2: Very good really, in a way. It's helped, but what I'd like is feedback from you. So, after the test, how did we do? I think the link is missing. You take information away but we don't know how we've done.<br><br>**I2: That's really useful, yes.**<br><br>R2: If there is something…<br><br>**Yes, we can actually feedback.**<br><br>R2: Yes, some ways we can improve.<br><br>**Well, surely since you did your assessment last week, I'm supposed to give you a phone call this week for follow-up and then I'll give you feedback.**<br><br>R2: No, that's provided by everybody; you have feedback and say, 'Well, I need to improve in that area. I need to…'<br><br>**We usually provide that in a phone call a week after the…**<br><br>R2: Yes, it is a feedback both ways really, and knowing: are you developing?	**Feedback on assessments' result is suggested**

McGowan transcribe + translate

**I2: Excellent, that's a really good suggestion, yes.**<br><br>**Yes, that's a good point. If I ask you all: did you discuss or talk about your participation to another participant in the programme? Do you know any other participants? P4, no. Christine?**<br><br>R3: No.<br><br>R1: No.<br><br>**You don't know anyone?**<br><br>R2: No.<br><br>**All right, so the last part of this session, we just want to talk about how feasible the programme was. If I ask: was any of the programme, any part of the programme - either recording diary or education session - were any of them difficult for you or you feel like that was useless? P4?**<br><br>R3: No, it was all really, really good. The one thing that I would like to do is something to assess my walking stride - just because I feel like sometimes I walk slower on my left leg than I do on my right leg, which my left side has been affected by. So, it'd be good to be able to measure my walking stride and analyse it to see what I'm doing wrong and how I can improve it.<br><br>**Good. You know, P4, we are assuming that because of COVID and people participating from other parts of the**	

country, it won't be feasible to watch the stride, because stride needs a very close measurement for that.

R3: Yes.

So, we were using some global walking assessment that…

I2: But happy to help, yes.

Yes, we are happy to help you with that.

R3: Yes.

I2: Yes, so in the next goal setting session, Ahmed can teach you how to measure your own stride.

Yes.

R3: Okay.

I2: So that you don't need us; you can do it from now on, yes?

R3: Yes, that'd be great.

I2: It's good that you are raising these issues, yes.

Mr P2, was anything difficult or you feel that…

R2: [Over speaking 0:48:59.3] with the start it's very difficult [unclear words 0:49:02.4] with the programme forward. A way I think it could be improved is maybe having a medical person

advising you, saying… If my left leg is not working and the right one is doing better, having a medical… I don't know, could we do an improvement on this? Like, have a medical… Okay, if I'm finding it very hard or not moving, maybe medical…<br><br>**Intervention?**<br><br>R2: Intervention can help, yes.<br><br>**Yes.**<br><br>**I2: Yes, it's more like having an inter-professional group working on such programmes rather than just the physios, really.**<br><br>R2: Yes.<br><br>**I2: Or having the facility for professionals leading the programme to refer you on for some more help.**<br><br>R2: Yes, I think something, a third eye: why am I having these problems? Is there some sort of - but again the programme is saying if you do exercises you will get better - which it does - but as you're going along you're getting those doubts, 'Why I am not doing this?' Just knowing. This is me talking about…<br><br>**I2: Yes, absolutely.**<br><br>R2: Why? I would question, 'Why am I…' It could be a medical reason. They've probably already done a study on it anyway.	**Having a physician within the team for medical advices**

Not knowing is it something - we need some sort of feedback on that, a third eye.

I2: Very good, valid point: people just don't focus on walking; they have other issues as well.

R2: Yes.

I2: If there is a need that is non-physical, we need to have another professional who we can play to.

R2: Yes, something to improve it moving forward.

I2: More holistic thing.

R2: Yes. It's not going to be perfect. It doesn't matter; you can widen so much that it's too wide, but trying to keep it into some sort of focus with a third person that could be medical or could be anybody else, you know?

I2: Excellent, yes.

So, let us move to the next question. It's about: how do you think this programme can fit with other services provided by NHS, if you have been receiving anything? So, how does this programme fit with other physiotherapy services provided at home or in the community? P4?

R3: I think my experience is different from anybody else's just because I didn't get that much physio. I would go to physio but we did the very basic things like walking up and down for a little bit and then that would pretty much be it, and testing my

responses to picking up things. Then she would give me examples of exercises that I could do at home by myself. I was like, 'But I need a little bit more help on what to do and how to do it.' Yes, it's just been really helpful. I can't fault the programme at all.<br><br>**I2: What about you, Christine; how did it fit into the other therapies that the NHS gave you?**<br><br>R1: Well, my NHS therapy was brilliant. As soon as I came from hospital, I had what they call the hand lady come because I couldn't move my hand at all when I got home. She helped me with lots of exercises for that and then got me - and then another physio… I had three different physiotherapists visit me, which was very good. So, therefore the programme has followed on from that so it's been just a continuation for me - which has been brilliant really, thank you, yes.<br><br>**I2: Thank you, Christine. How did our programme…**<br><br>R2: It was mixed up for us. We're able to do things because you - we are lucky. As soon as you - lucky to have it and giving motivation to us, like showing you how to do… Just showing you that we are doing it right or wrong, in this way it has helped me anyway a long way. Otherwise I don't think I would've, if they just say, 'Go away, do this and do that.' Having a physio was a very important factor, I think.<br><br>**I2: So, the NHS gave you some physio?**<br><br>R2: Yes.	**The programme was a continuation of what received from the NHS**

**I2: Then how did this…**<br><br>R2: Well, it's linked into it, you know? You're getting a second idea. I'm very lucky to be in the position I am at the moment - especially because people around me are giving me the motivation to move forward, trying to be more active. We have days where you don't, where you are just not in the mood or lack your body energy. It's just important as well, but physio has helped a great deal. If we didn't have the physio, I think I wouldn't be in this position.<br><br>**I2: Very good, yes.**<br><br>**So, if I ask you moving forward: when you're finished with us, with this programme, are you planning to continue doing the same thing, setting goals and trying to do your continuous training? Can you let us know a little bit about the plans? P4?**<br><br>R3: I want to be able to do some dancing aerobic exercise; that'll be fun. I have to get to a point where I can get up the stairs [laughs] and not be still exhausted first, so it's definitely helped doing that. I want to be able to do some sort of martial arts [laughs]. I'm going to try some basic just - I think it's helped because it's trying to improve my balance. That has been my major thing - and I know that martial arts is very much balance and coordination, which I still struggle with, but I'm definitely going to be working on that more this year.<br><br>**I2: Brilliant.**<br><br>**Christine, are you likely to continue?**	**Motivated by the programme**

R1: I'd say wow to that [laughs]! Well done P4, yes.<br><br>R3: Thank you.<br><br>R1: Yes, well done. I shall continue to try and motivate - well, yes, to motivate myself. Of course the family will be sorting me out, getting me to do more, but yes, I will certainly want to improve.<br><br>**I2: So, you will be setting your own goals and doing exercises?**<br><br>R1: Yes, that's right. I do at the moment still suffer quite badly with fatigue, which I've found a difficult part of the stroke. I've heard about strokes over the years but I didn't realise quite what - how the fatigue can affect you. It's just even your arms are tired and, yes, it's just… I just can't do anything for a while during that time. I find that very difficult, but the rest of the time I manage to motivate myself into trying to improve and will continue to do so.<br><br>**I2: Brilliant. I have to tell you, Christine, you're not alone in this; most stoke patients have post-stroke neurological fatigue. Nobody knows how to sort it out, but it's just a…**<br><br>R1: Yes.<br><br>**I2: When you're tired, rest - but when you're able to, push yourself.**<br><br>R1: Yes.	**Fatigue affects capability for SM**

**I2: So, you're not alone in this. It's a very common syndrome, post-stroke neurological fatigue.**<br><br>R1: Yes.<br><br>**I2: It will get better; people tell us years down the line they get better.**<br><br>R1: Get better, yes.<br><br>**I2: Yes, we have service users who tell us they used to be really tired, but ten years down the line they're like, 'Okay, once in a while; not as frequently as before', so things will get better.**<br><br>R1: Oh good.<br><br>R3: Ten years down the line [laughs]?!<br><br>**I2: I am not telling you to wait for ten years [laughs]. No, P4! No, I'm just saying the person I am talking about is ten years down the line and he says, 'Yes, I am getting…'**<br><br>R3: Okay.<br><br>**I2: He doesn't notice the fatigue anymore, yes, so what about you, sir? Will you be continuing the programme?**<br><br>R2: Definitely I will continue. My aim is to lose weight; I have put on weight - which I'm not happy about - but my goal is to	

lose the weight. I don't know how. Again exercise, but nothing like martial arts; I don't know about martial arts [laughs]!<br><br>R3: That'll be a couple of years down the line!<br><br>R2: Yes, but a very simple programme to get my weight down to where I am happy. At the moment…<br><br>**Yes, just go to the park as much as you can.**<br><br>R2: Yes, I am lucky; I have got a gym and I'm a member of the gym anyway, but sometimes you have days off, you know? You can do so much - and the next day, you don't want to do anything, but knowing your own limit is very important, I think.<br><br>**I2: If you figure out how you do that, please let me know. I still have not been exercising since 1st January so I need to…**<br><br>R2: I use the gym every...<br><br>**We just want to see if you have any comments - moving to another question - about the way of our communication or approaching you every week or every other week; the way how we can set meetings and appointments. Is there any comment? Feel free, please, to let us know.**<br><br>R3: No, I found that text message is easier than email sometimes, because sometimes I don't get my email notifications come through and I have to physically go into my email to check if there is a new email. So, if you even just text me like, 'Check emails', then I'd be like, 'Okay, cool.'	**Desirability to continue**

R2: I think I would agree with that. If you can do both that's even better, but I find text easy, you know?<br><br>**Christine, any comment on communication?**<br><br>R1: No, all been good for me, thank you.<br><br>**I2: I was hoping you would say, 'Ahmed, you've been pestering me so much for the past three months!' [Laughs].**<br><br>R1: No, all good for me, thank you! Yes, all good for me.<br><br>**I2: Thank you, Christine.**<br><br>**Thank you. The last question - and we will end there - just: is there any way we can make things better? If you have any idea, any comment how we can make it better, please. P4?**<br><br>R3: For me, I'd probably want to have a few more exercises that are a little more strenuous - but just for me. I find sometimes the basic ones are easy enough that I can do them without thinking about it, so a little bit more intense would be good.<br><br>**All right, we'll consider that.**<br><br>**I2: Yes, that's another thing Ahmed will put on his list. He'll do some research and he'll download some more harder exercises for you, P4. For your balance, Tai chi has got a great amount of evidence. If you find somewhere that does Tai chi around where you live…**	**Different ways of communication are suggested based on participant's preference**<br><br>**Happy with communication**

R3: Okay.<br><br>**I2: …Tai chi, join those classes for a couple of months and you will see the difference because it's like a great amount of evidence for your balance. Ahmed will give you more strenuous exercises - even though it's not within the programme. It's about tailoring the programme, so yes.**<br><br>**I think she still has some challenging exercises with the exercise booklet that we…**<br><br>R3: Yes.<br><br>**So, we might move to advanced a little from next week. I will call you later.**<br><br>R3: Okay, no problem.<br><br>**Yes, we'll discuss the details.**<br><br>**I2: Brilliant. Do you have any ideas for us, Christine, to make it better?**<br><br>R1: No, not really. No, the exercises have suited me fine. As I say, being an older participant, maybe that makes a big difference, but no, not really. No, everything has been brilliant, thank you.<br><br>**I1: Thank you.**<br><br>R2: Yes, well, the programme has been very good in getting us, I think, to the level where I am at the moment anyway. I think	

the next stage is, where do we go? With [unclear word 1:03:48.3] and that sort of thing, that's where the guidance will be good for us, still, to move into the next level or something.<br><br>**Sure.**<br><br>**I2: Yes, we will guide you.**<br><br>R2: I think that would be a good thing, yes. Your general health, really.<br><br>**I2: Yes, we'll guide you.**<br><br>**We'll finish there. Thank you so much, everyone, for your time.**<br><br>**I2: Anything you wanted to say - we're going to [signal breaks up 1:04:20.0] you - but you can always keep in touch with us and…**<br><br>**We'll stop the recording for now so you can just feel free if you want to add…**<br><br>**[Recording ends mid-sentence]**<br><br>**[END OF TRANSCRIPT]**	

All right, thank you, and you are okay with recording, right?

Yes, I've got a picture in front of me of writing, can that be got rid of?

What's that, sorry?

There's a piece of writing. It said, 'This meeting is being recorded, ' can that be taken off the screen?

Yes, just keep it as it is. It will go in time so don't worry.

Okay.

So, P5, P7, start please.

M2: [?James Simpson], sorry, what else did you want to know?

The recoding, you agree to record the session?

M2: Certainly agree to the recording, and I hope it serves people well.

Thank you very much. I'll just remind you, guys, feel free to accept if our thoughts you want to share with us, feel free, please. Don't worry, there is no wrong or right answers, or don't feel like you don't want to bother us with any free explanation. So please, just tell us freely what you feel about your participation. All right? So do you have any questions before we start?

Who are the people that are going to participate from the other side rather than our side?

No, it's just, P5, P7, [?Sheeba] and me. This is this meeting and it will be doing a couple of meetings similar to this with other participants in the study. So we already have done two and we'll be doing some more in future for every participant possible and who agree to take part in the focus group. So it's just you and P5, P7 and Sheeba.

Transcript	Codes	Themes
**All right? So start, guys, with the first question. Can you just tell us please why did you agree to take part in this programme?**<br><br>Who do you want to speak with, me?<br><br>**I2: James, do you want to go first?**<br><br>M2: Yes, I think the one that comes to mind is, hopefully, we can pass information over that's benefitted us and hopefully benefits other people.<br><br>**In general do you think the programme was useful for you?**<br><br>M2: Yes, definitely.<br><br>**I2: [?P6], why did you agree to join the study, P6, when we approached you in the beginning?**<br><br>Well, one of the problems I've had for a while because of the	**Reasons for joining the programme (to pass information that help us and other people)**	**Motivations**

McGowan transcribe + translate

strokes, has been the balance, and because you were doing a research-type programme on balance. I considered that you would be looking at things slightly different from the normal people, which may mean that you find something that they haven't, that helps me.<br><br>**Do you think the programme was useful for you?**<br><br>Yes, I did actually. Most of the programme was okay. The only problem I had got was I couldn't do the stand-up balance parts properly. So I don't think I got the full benefit out of that. I had to hold onto something.<br><br>**So let us move to talk about the programme, like elements, so starting. We have done a couple of things since you started with us, so at the beginning we did assessment and then we did an education session over Zoom just talking about safety tips, and how can you do training. Then we did the programme for 12 weeks**	**Reasons for joining the programme (to improve balance)**	**Motivations**

**including exercise and a group session every couple of weeks. So let us talk about each element of these elements. So starting from the education session that we did over Zoom for everybody, so, P5, P7, if you can start first. To what extent was the education session helpful for you?**<br><br>M2: Well, you were in an environment where there were people that have been very similar to yourself and you could talk, and whatever, and yourself and Sheeba as well. I just think it's been a huge help to me. Although I've got up and come down and I'm going back up again, but without you people and the physios, I think that I wouldn't have been as far now. It does give you a little bit more confidence. I've started to walk again around the road. I fell over, got myself back up with the help of the lads and lasses that are there, and I just think I've come quite low. People keep saying to me, they say, 'P5, P7, you're walking.	**Huge help to participants**<br><br>**Appreciation of help**<br><br>**More Confidence to walk**	**Participants' evaluation of the programme**<br><br>**Perceived benefits**

You couldn't walk when you left the hospital,' blah-blah blah, and I think it is useful; very, very useful.<br><br>**P5, P7, do you still remember on the first week when you joined us that we did the education session, talking about safety tips at home and also how this programme will help your confidence to do training alone at home?**<br><br>M2: Yes.<br><br>**So how easy was it to understand the information provided in that education session?**<br><br>M2: What, to work on my own after?<br><br>**Yes, how was the information that we presented last [over speaking 0:08:07.7]?**<br><br>M2: Yes, well, we were all starting there, weren't we? Yes, the information was obviously good and I haven't spoken to		

McGowan transcribe + translate

one person that I don't particularly… I get on with them and it's very good the way I think your programme is run. So yes, I think it's been a…<br><br>**P6, can you tell us about the education session that we did at the beginning when we presented safety tips for your training? To what extent was that session helpful for you?**<br><br>Well, the main thing about that was the understanding of what you were offering and what was wrong with me, <u>because if I have an understanding of what's wrong and what you're offering, I can coordinate the two and it makes more sense then</u>. So when you're doing the exercises you're doing it for a particular reason and you can see then whether you're developing or not. When it doesn't go right, which has happened with me since, I have a better understanding. So you don't actually give up because you've got a purpose and a goal to aim at, and that's because of the	**Delivery of the programme was very good**<br><br>**Education session helps ability for SM**<br><br>**Education session and goal setting work together to support continuity of therapy**	**Participants' evaluation of the programme**<br><br>**facilitators of SM/Perceived benefits**

support that you've been given from this understanding.<br><br>**How easy was it for you to understand the information that we present at the beginning in the education session?**<br><br>It was pretty good, actually, because I've got a reasonable medical history and I'm also a chemist. So I could understand a lot of the things anyway. When you apply them to me then obviously it made more sense because, knowing things and not knowing where they apply to you, that's not quite the same. So I had a good understanding and the exercises and the mobility. They check your brain, that sort of thing, I thought that was really good. The girl actually said I had a 'Brain activity of a 60-year-old,' which I thought was quite good for somebody who's 35! Eighty-seven, actually.<br><br>M2: You don't look that old.	**Easy information of education session**	**PArticipants' evaluation of the programme/Perceived benefits**<br><br>**Participants attitude**

Pardon?<br><br>M2: You look younger!<br><br>You're trying to be my friend, aren't you?<br><br>M2: Yes!<br><br>**So can we just move, guys, to another aspect, to talk about the other component of the programme where me and Sheeba visited you at home to set goals for your programme and also provide you with the sheet and pedometer and exercise booklet. So what do you think of the structure and content of the intervention as a whole? P5, P7?**<br><br>M2: Me, yes, very good. Obviously you give us the pedometer and one thing and another, and give us things to do. I think the programme is it's good. It certainly helped me and I'm really, really hopeful now that I can press on. Like P6, he's had setbacks, I've had a couple of setbacks, not as bad as that,	**Structure and content were very good**	**Participants' evaluation of the programme**

but as P6 just said you'll still be up. He'll be up and running soon, not literal, but yes, I just think everything that's been on the courses have been helpful.<br><br>**P6, what do you think of the structure of the intervention?**<br><br>Well, I understood and thought the structure was very good, but I think I had an advantage that with the information that you were giving and the instructions, and also the one that the stroke people were giving, a lot of them were parallel. So I was doubling up, if you like, on the exercises and getting explanations from two sources. I used to have the physios from the stroke come to the house and go through them and then I had yours as well. I think the big thing about all this and the person I am, it creates a motivation when you know things are not going to become as perfect as you would like them to be.	**Everything has been helpful**<br><br>**Providing support from 2 different sources (NHS and our programme) creates motivation**	**Participants' evaluation of the programme**<br><br>**Participants' attitude**

**How did you find your involvement in setting the goals, and planning the activities for your walking recovery with Sheeba?**<br><br>**I2: I think P6 must have forgotten, like we sat together and we made goals for you on Zoom.**<br><br>**With P6, you did online on that day?**<br><br>**I2: Yes, on Zoom. Yes, I didn't go to P6's house. I was just thinking P6 is the only person I didn't go to his house. You didn't invite home, P6!**<br><br>Pardon? Say that again, I'm sorry?<br><br>**I2: I said, 'P6 is the only person whose house I didn't visit.' I said, 'You didn't invite me home for your goal setting.' I set your goals on Zoom, didn't I?**		

That's right, well, some of the goals I did achieve and some of them I know I won't achieve. So I still aim for the same thing, but we had one goal where I had to go a certain distance in a certain time, walking that is, and that's I don't think that's ever going to be achieved now.<br><br>**P6, how did you feel about your involvement in setting goals and planning for your exercise?**<br><br>Well, I like to aim at goals and things like that. I'm told I'm quite awkward that's why I've survived so many things, but I have to overcome the thing that's handicapping me if I can. So with these exercises it gives me if you like, the instruments to try and overcome the incapacity.<br><br>**P5, P7, can you tell us how do you feel about involvement in goal setting and action planning?**	**Self-motivation helps SM**	**Facilitators of SM**

M2: Involvement in what, sorry?<br><br>**I2: Setting goals with me, you know we sat down together and we discussed what is important for you, very important for you?**<br><br>M2: Yes, to walk as normal as I possibly can. I'm not as bad as P6, but the balance that's a bit of a problem for me but I think [?George's] got to iron that out, hopefully. Yes, it's still not…<br><br>**I2: Did that help, P5, P7? Does that help? When I came to your house and we sat and we discussed and filled the goals in your booklet, did that help thinking about your goals? What do you think?**<br><br>M2: Yes, well, that's why obviously we set them, don't we, and you try and achieve them, but I just… I must admit, for about a month, six weeks, I was really on a downer, but George, he's a good lad and he really is pushing me. So and		

obviously I'm as pleased as anybody to be pushed and hopefully see more light at the end of the tunnel.<br><br>Can I just mention one thing?<br><br>**Yes, please.**<br><br>I fell over quite badly and it took two men to pick me up and straighten me up, and the only thing they asked me was had I 'Been drinking?'.<br><br>M2: Christ!<br><br>I'm teetotal.<br><br>**I2: What a shame, oh, my God.**<br><br>M2: Oh, God, no, that's…<br><br>**This is unethical I think.**<br><br>**I2: It's terrible; terrible; sorry, P6. Sorry to hear that them people have been mean. This is actually being mean to you.**		

Well, I've started drinking diluted water now to make sure!<br><br>M2: You're sure it's diluted water and not vodka?!<br><br>Incidentally, Sheeba, you're more than welcome to come and visit me.<br><br>**I2: All right, definitely, next time, right gentlemen?**<br><br>**For assessment, yes.**<br><br>**I2: Yes, I come home to see you.**<br><br>Unless you prefer older men.<br><br>**I2: There's no preference, P6, I'm coming to assess you, all right?**<br><br>Yes, I'm being a bit naughty now.<br><br>**I2: Yes, you're being cheeky. [?Ahmed 0:17:44.2], do you want to continue with your exercise questions, sorry?**		

McGowan transcribe + translate

**Yes, so the question, what were the difficulties when we set your goals? What were the difficulties in setting your own goals?**<br><br>Are you talking to me now?<br><br>**To both of you, you can start if you are ready.**<br><br>Well, there's certain goals I had to tick. One was trying to walk more and I had an extra goal, if you like, because of the second stroke where the nurse said I 'Hadn't got a stroke,' the operation of my hand is not as good and it took me half an hour to tie a shoelace. So one of the goals that I was doing with the stroke people was to manipulate my hands to try and improve that type of action, and then you've got the thing where I had this jumping of my legs. I think they weren't sure whether it was lazy-leg or what, but then that was causing problems as well, because you would be sitting down and suddenly your leg is jumping up and down and		

you can't stop it. So you've got things like that where you have to try and find out to stop it. What I found by trial and error was ferrous fumarate seemed to calm it down very quickly, and so I achieved something there which was not in the programme, the thing.<br><br>**P5, P7, can you tell us about difficulties when you were setting goals?**<br><br>M2: Obviously the walking became a problem, and before, I was doing six, 7,000 steps a day and I thought, I'm going to crack this not a problem. Then all of a sudden COVID and I wasn't walking as good, and I don't know whether P6 would agree, but you do lose your confidence.<br><br>Well, you do because you're forever falling over and you don't actually know you're going to be giddy or fall over. You just suddenly tier and that's it, and you can't stop yourself.	**COVID causes loss of confidence**	**Challenges**

M2: Yes, fortunately I've only fell over in the house and I fell onto the settee but, so that's, it's a nothing as such.<br><br>No, I hit the guttering when I fell over.<br><br>M2: Oh, blooming hell, I see, that's not good.<br><br>What I've done, though, and this is not good, but I've bought myself an electric scooter which folds up. So theoretically I can walk with it a bit and then get on it and have a ride.<br><br>M2: About two months ago I was thinking about getting one of the whirly things, the scooter.<br><br>You can take them on buses as well because that saves the battery if you want to go further.<br><br>M2: Well yes that's true, but in the end me and my wife we said, 'Look, you're going to get better,' and eventually after having various things I'm more	**Using electrical scooter can help goal achievement**	**Facilitators of SM**

McGowan transcribe + translate

positive now. I really am, but I think a lot to do with that is George. George has been great for me. I've only had him two or three weeks, but he's great.<br><br>No, I had him for six weeks. Have you got a walking frame?<br><br>M2: Who, me?<br><br>Yes.<br><br>M2: No.<br><br>No, well, that's the thing that helps you to stand up straight and walk with something to hold onto to give you the balance encouragement.<br><br>M2: Yes, but unfortunately, P6, I'm a bit… I don't like to use anything especially if I'm walking outside with a stick. I hate it because if I see anybody…<br><br>I swore I would never walk down the street with a walking frame and I did.		

M2: Yes, I understand that.<br><br>All the traffic stops for you when you cross the road and I give a royal wave, and all that stuff so you get used to it.<br><br>M2: Yes, you've got to, that's the way to answer it, isn't it?<br><br>Put your pride in your pocket, it's what does you good that matters.<br><br>M2: Yes, but as I say, I'm just so glad that I'm back and walking. It just makes you feel much better in yourself as well when things are going a bit right. I'm not saying I've got everything right, that's wouldn't… It's not the case but…<br><br>**So can we move a little bit to talk about, you remember at some of the group session that we do every two weeks over Zoom? So at some point we invited some senior patients, like the judge, I think her name is…**		

M2: Like the girl, the woman?<br><br>**One time… Sheeba, what was the name?**<br><br>**I2: [?Brynn]. Brynn came over. Yes, Brynn is a teacher with the stroke for past so many years. He came over to speak as well.**<br><br>**So what do you think of having peer or carer support?**<br><br>**I2: Expert service for stroke patients talk to you.**<br><br>**Yes, while you are carrying or doing the programme, what do you think of that?**<br><br>M2: I think any… Well, you've got to make your own judgement. If somebody's going to speak and you've got to make your own judgements on it. I would imagine, the majority of people will give you good knowledge of what's goning on and I saw the lady that was…	**Liked meeting with peers in group sessions**	**Participants' evaluation of the programme**

What's her name? She's from down south.<br><br>**I2: Yes, [?Liz], the judge.**<br><br>M2: Liz, that's it, that's the one. I thought she was really, really good. You felt encouraged by her and I really do think that those sorts of people, they give you confidence.<br><br>**I2: P6, did Brynn speak to you? Was Brynn in your role?**<br><br>What was the name?<br><br>**I2: I think you were with Brynn. I don't think you came for Liz's session. Brynn [?Halliwell], the teacher.**<br><br>I don't remember that actually. The only thing I remember is that you gave me the name of a man that we spoke to in one of these sessions and he never answered me.<br><br>**I2: Did you write to him?**	**Meeting with peers really good**<br>**Peers support enhanced SM**<br>**Increased confidence**	**Participants' evaluation of the programme**<br><br>**Facilitators of SM**<br>**Perceived confidence**

I sent him an email that Ahmed gave me his email address, and then I think he contacted him as well and he never actually replied to me.<br><br>**I2: Okay, we can chase it up for you if you want?**<br><br>Well, the thing I was interested in was she was saying, and this is another thing of understanding what's happened to you. They've got a new process for treating strokes now, where instead of thrombolising it they actually suck it away, and I was quite interested in that because I thought it should decrease the damage that affects your brain, but I never got the [unclear word 0:25:28.3] with that.<br><br>**P6, can you tell us how did you feel after meeting that person in the group session? Was it helpful? Was it a good thing?**<br><br>When you spoke to me on the Zoom?		

**Yes.**<br><br>I thought that was quite interesting, but then I wanted to take it further and you gave me the email address and he never answered me.<br><br>**I2: We can chase it up, though, can't we, Ahmed? We can say, yes. We meet him quite regularly. Yes, carry on, Ahmed.**<br><br>**So if you just tell me how was the group session that we did every two weeks doing the exercise with the group or for Zoom? How was it do you think? P5, P7?**<br><br>I thought the group sessions were quite good except sometimes you couldn't see what the person is doing. Remember, there was one lady and all you could see was her head bobbing up and down. So you couldn't tell what she was doing against what you were doing, and sometimes I know I	**Disadvantage of online sessions** (sometimes you couldn't see what the person is doing.)	**Challenges**

found this when I was with that George, you're told what to do and you don't interpret it properly. I don't know if you can understand that?<br><br>**Yes, P5, P7, what do you think? How was the group?**<br><br>M2: Yes, there's a lot of nice people on there and you could feel the warmth as such and, yes, it was I find everything I've done has been helpful, some more than others, but yes it's…<br><br>**Overall, were you happy with doing exercise with the group or wasn't that happy for you?**<br><br>M2: What, working with the group?<br><br>**Yes.**<br><br>M2: Yes, I was in hospital with [?Steve] for nearly six weeks, so it was nice to be with somebody that I knew before, but it was just nice to see other people as well and getting on. I think	 <br><br><br><br><br><br><br>**Happy with meeting people in group sessions**	 <br><br><br><br><br><br><br><br><br><br><br><br><br>**Participants' evaluation of the programme**

that's a bonus. If you see people…<br><br>Can I say something?<br><br>**Yes, [over speaking 0:27:58.3].**<br><br>It was all the exercises you we're giving, I found them quite easy for the simple reason that before the stroke I was going to the gym for 12 years before that.<br><br>M2: Christ!<br><br>I used to go on a regular basis. People used to laugh because of my age, but I still thought it was worth doing.<br><br>M2: Christ, yes, it doesn't matter how old you are, age is a nothing.<br><br>Eighty-seven, or to the lesser now I'm 35!<br><br>M2: You don't look that old, 35? Phwoar!	**Engagement in exercise pre-stroke helps SM of exercise post-stroke**	**Facilitators of SM**

McGowan transcribe + translate

They don't believe that. I can't get it across to them.<br><br>**Guys, I want to ask you about the recording diary that we have given to you so and the pedometer. I know some of you they have done their best and some didn't do it at all. Some have completed the full record. So regardless of that, can you just tell me how did you find doing your exercise at home by yourself without having us as physios to visit you, also, evaluating your performance without the therapist?**<br><br>M2: At first you think you can… I've got to choose my words right here. You think you can get away with some and do others, but that's not the case. What you need to be doing is basically everything. I'm not saying I cheated, but I thought, I would do more of say something else and than what we've got, but yes, it catches you out because you're not as speedy as a recovery. I think the		

fitness programme was good. As P6 said, it was a bit easy for him because he had done various things before and you could appreciate that, but in general I think it was good.<br><br>**So did you think for people after stroke in a similar situation, they can carry out the programme on their own, or did you feel at some point that you need some support from a physio to visit you at home to do the exercise with you, or to evaluate you, or you felt it was okay?**<br><br>M2: Well, it's how badly the stroke's affected you and how badly you want to do it and if…<br><br>I think you do need support, though, no matter how you feel, you need someone who actually you feel is looking after your interests, and so somebody coming and helping you and watching you does make a big difference.	**Having a carer helps SM and recovery**	

M2: Yes, in that respect, but I just look at myself now and like P6, he's had some ups and downs, and as I say before to a lesser degree I have but we've got to be a bouncing ball. We've got to bounce back, that's the only way.<br><br>Yes, but with somebody coming to support you they do help you to get up and have another go.<br><br>M2: Yes, totally, yes.<br><br>Sometimes I think you can give up on exercises, and if somebody comes back and helps you to get back onto it you've got that encouragement, because it's hard.<br><br>**Well, you know guys because of the pressure on the NHS services so it might be hard to cover the demands for everybody by providing physio to come every week or every couple of days. So this programme is part of covering for the shortage in the services for people who can manage**	 <br><br><br><br><br><br><br><br><br><br><br>**Follow up was encouraging**<br><br>**Follow up can keep you going with exercises**	 <br>**Facilitators of SM**<br><br><br><br><br><br><br><br><br><br><br><br><br><br><br><br><br><br><br><br>**Participants' evaluation of the programme**<br><br>**Perceived benefits**

**for themselves. So they can be supervised just slightly by us every other week or just giving a call every week to see how you are doing, and then we ask people to carry out and to evaluate their training every day on their own. So based on that, what challenges did you guys face while you are carrying out the programme?**<br><br>I'm sorry, I didn't hear that really.<br><br>**What challenges did you face during the programme?**<br><br>Well, the big one for me was the walking because in the beginning it wasn't very good and it gradually got worse. So it became harder and harder to do, walking any distance, and then eventually it became harder because you've got the balance problems. I had to take to the walking frame to actually do the distance anyway.	**Walking wasn't very good at the beginning**	**Challenges**

**Yes, but if you feel like safety was a challenge at some time or you were able to manage your safety at home?**<br><br>I do manage it myself. I've had quite a few things where I have to be independent so that helps. So with the walking I could go out on my own with a walking frame, knowing that I wouldn't fall over and I could do more distance then.<br><br>**How about you, P5, P7?**<br><br>M2: As I say, the first three months when I got home, I was flying, absolutely, and COVID stuck which is obviously a different thing to what… Various other things, but I'm just… If I took all the credit for bouncing back I would be a liar. I think my wife has to take an awful lot of credit. She does everything, honestly, and she won't let me… She's on the ball all the time and I'm so grateful.<br><br>**So this is our next question about what helped you to**	**Wife was a great supporter and care**	

**participate in the programme to the best of your ability, so maybe your wife will be the first thing to be helping you with participation?**<br><br>Yes, true, yes she could, but in all fairness, I wanted to come on just in case you could help anybody else. You might think you're saying something and it's just second nature, but it might be helping somebody. I don't know.<br><br>M2: Sorry?<br><br>**I2: James, I have to say, when you said about your wife, she was so good at organising she would respond to our calls. She would fix you appointments. She had made sure that all the processes in the study, they happened on time. She would push you to do the exercises. So I totally agree, you have a very, very supportive family who…**<br><br>M2: Yes.		**Facilitators of SM**

**You are lucky, P5, P7, not everyone does, that.**<br><br>M2: I know, yes, but I'm scared of her anyway so…<br><br>**I2: P6, what facilitated you? What pushed you to do well in this programme, or just like on Monday you thought, oh, I have to do my exercises; why? What made you motivate yourself to participate in this self-management programme?**	**Having enough time for adjustment can help SM**	
Well, I have quite a lot of time on my own, actually, so fitting things in like that is not a problem. In fact, I have too much time. So a lot of it I can do as a matter of something that's passing the time away if you like. So it acts in two ways, but then along with this, I've got the stroke people coming and you're doing the same thing twice. I think the other thing about programmes like this there are people who don't get this sort of programme. So when they come out of hospital	**The programme provides great help after leaving hospital**	**Facilitators of SM**

they regress because they don't know what to do if that makes sense.<br><br>**I2: Yes, that's very true, thank you.**<br><br>**So can we move to another aspect of the programme to talk about the methodology? So, guys, you know when you were selected and you were the lucky group to have the programme, because everyone who's sent to us by the NHS were randomly assigned to one of two groups. So the one group that you're assigned to, called the 'Intervention Group,' where you had an education session and then you had the opportunity to set your goals. To have the pedometer and the evaluation for yourself, other groups they only received the education session and then we didn't do further help for them. This suggests the way to evaluate the effectiveness of the programme that we are running. I just want to ask**		**Participants' evaluation of the programme**

**you, what do you think of the design of the study, like, that some people will be assigned to intervention group and some people will be assigned only for the group with the education session and no further help? What do you think of that?**<br><br>Can I say I don't believe or don't like placebo effects, because I think you're killing one lot and that the other one doesn't know whether they're being treated either. So you're not really sure whether you're being treated properly or whether you're just being left to let it happen as it happens. So I always think these programmes should be much more positive. I had an aortic aneurysm and that was done on a placebo basis. You picked one or the other to see which was the better, and in my own mind I thought the one which was more modern, is a much safer one, and so I made such a fuss and I got on that. With the other ones you don't really know because it's normal	**Didn't like having a placebo treatment in the study**	**Participants' evaluation of the programme**

or it's not normal and you're not being treated at all. I don't like that.<br><br>**Okay, and what do you think, P5, P7?**<br><br>M2: Very similar, yes, if you've got two groups surely they should have the same.<br><br>I think you can have two groups who have different treatments, but they are treatments and you work out which one is the better one, but not there's one who's not having any treatment at all.<br><br>**For just explaining to you, most of our participants they are receiving or they have received some help from NHS physios. So our programme was an addition to what they're already receiving from the NHS. So we are not just ignoring people, or just leaving them for nothing. So we just ensure they already have been receiving something or have received some help from NHS physios, plus what**	**Everyone should has the same intervention**<br><br>**Comparing 2 effective treatments instead of no treatment in a group**	**Participants' evaluation of the programme**<br><br>**Suggestions**

**we have provided. If we knew it is only us providing the help then it is a shame on us to leave them without any exercise planning.**<br><br>Yes, but I looked on your programme as being more intensive than say, the NHS, because you're on a research-type project. So you would be putting forward ideas and things that are not necessarily accepted, but they could work and you are trying them out for the benefit of the patient. So I actually thought in my own… So I answered it straight away when there was an application, that you have a chance of being given better treatment in that respect than the NHS. Is that right or not?<br><br>**Yes, and do you think they used outcomes to reflect what is important for you to obtain from rehabilitation? Like the thing that we assist every time about walking speed and confidence in doing your activities at home. Do you**		

**think the outcomes that we are using reflect what is important to you in your daily life?**<br><br>I'm sorry, I didn't quite catch the question, could you say it again?<br><br>**Yes, the thing that we did during the assessment, like walking speed, measuring your walking speed and a number of steps per day, do you think these outcomes are the things that you are looking for out of your recovery or not?**<br><br>**I2: Are they relevant to you? Were they use'…?**<br><br>Yes.<br><br>M2: Yes.<br><br><u>I thought the pedometer for instance, was a relevant thing because it was giving a measurement, where in some cases you wouldn't have bothered</u>. I couldn't understand	 <br><br><br><br><br><br><br><br><br><br><br><br><br><br><br><br><br><br><br><br><br><br><br>**Outcomes were relevant to what participants are looking for**	

it but sometimes I did quite a high walking distance, and other days when I thought I had been busy I had hardly done any walking at all. What I think, you encouraged me the other day, Sheeba, because I've got this scooter, that I should spend more time walking around the house.<br><br>**I2: Yes, I did tell you that. No, when Ahmed and [?Jessica] came to your house and they made you do walking. They made you do cognitive tests. They made you do all these assessments. Did you find them useful or relevant to you, or did you think, oh, this is such a waste of time, P6?**<br><br>No, you mean when the girl came for the assessments?<br><br>**I2: Yes, Jessica.**<br><br>Yes, no, I <u>thought they were very, very, good actually and quite sensible, and of course you had got the comparison of</u>	 <br><br><br><br><br><br><br><br><br><br><br><br><br><br><br><br><br><br><br><br><br><br><br><br><br><br><br>**Assessments were very sensible**	 <br><br><br><br><br><br>**Participants' evaluation of the programme**

the beginning and the end. So I quite agreed with that.<br><br>**I2: Yes, what about you, James?**<br><br>Sorry?<br><br>**I2: I'm asking James. James?**<br><br>M2: Yes.<br><br>**I2: Do you remember Jessica coming and doing two assessments with you?**<br><br>M2: Yes, back to the pedometer, I thought that was, to me, and I've played a lot of sports, I wanted to be… Let's say I had three, 4,000 whatever steps, the next day I wanted to get more, and I think that was a challenge in itself. I haven't got that now, but on my phone I can do the steps and the walking. It's something to… You want to be what you… It's impossible to start off at let's say 3,000 and every day you improve, else you would be crazy, but it's just an	**Using pedometer was good for challenging**	**Participants' evaluation of the programme**

extra shove I think anyway for more steps.<br><br>**Guys, about the programme and I want to ask you if you have discussed any of the exercise, your information that we provided during the programme to any of your friends or people who you are working in, like stroke groups or people who had a stroke like you?**<br><br>**I2: I think James… Sorry, P6, carry on.**<br><br>Sorry, I was going to say, I have expressed, well, expressed, I've told people about it and I've actually shown them some of the programmes so they could try them out. Some of them found it quite… Some of them were actually much older people and they found some of the sit-down ones very, very useful. I do tend to talk about it, I think, that sort of thing to people who I think it's relevant to.<br><br>**Who is this, P6?**	**Sharing the programme with other patients**	**Facilitators of SM**

Sorry?<br><br>**Who did you share it with if I can ask?**<br><br>Well, there were some people that I knew that had had strokes and, well, actually, there was one man which feels quite strange. He was a very advanced Parkinson's disease man in the ward I was in. I joined forces with him and we managed to communicate, and I was able to show him some of the exercises and some of the things that helped him become… Or feel more normal. I used to talk him into doing things because he could understand me verbally, but on his own he said half the time he 'Didn't know what he was doing.' So we made a communication bond if you like with the exercises. Some of the others were older people because I go to this place on a Thursday where a lot of them are… One of these places where	**Other patients benefited from the programme/ the protocol can be adapted for other patients**	**Participants' attitude**<br><br>**Perceived benefits/ suggestions**

McGowan transcribe + translate

they put old people to give the other people a rest.<br><br>**Respite.**<br><br>That's right, yes. Some of them they are way passed ending anyway, but some of them are interested when you tell them some of the things you can do. Recently I gave them, the carers, some of the Tai Chi stuff that they could do sitting down.<br><br>**I2: James, have you discussed this programme or study? I know you did it with Steve and you got Steve onboard, but did you talk about this to anybody else?**<br><br>M2: This is going to make you laugh and if my wife's in the kitchen, but my mother-in-law, she's 88, and she said, 'I don't worry about those little pedometers,' and she walks around her block, which is a good walk, and she does that virtually every day, and she's 88, with a stick. She went, 'If I can do it, you can do it,' and it	**Inspired by older relatives/relatives encouragement increase SM**	

was an encouraging thing. I said, 'Oh, you was moaning at me again,' but it certainly was and it was an encouragement. Eighty-eight years of age, [unclear word - signal breaks up 0:47:40.0], and she's unbelievable, an inspiration.<br><br>**I2:Ahmed, do you want to carry on?**<br><br>**Yes, the last part of this session and we'll be ending soon. So was any part of the programme including the education session at the beginning, the goal setting, the action planning, the group session every two weeks, and yourself, feedback and if I'm recording your training. Were any of these things hard for you to understand or to carry out every day?**<br><br>M2: Me personally, no, because obviously everything was done for us, apart from actually doing the activities.<br><br>**How about you, P6?**	**Having another problem can affect recovery post-stroke**	**Motivation/ facilitators of SM**

I did have quite a lot of problems later on because of this increase in the lack of balance. So there was a certain point of time, when there was some of what I would have said was simple exercises, and I couldn't really do them, not properly.<br><br>**Well, so the exercise was hard because you lost some balance.**<br><br>That's right.<br><br>**So how can we help do you think with these challenges? Is there a way you think we might help with that?**<br><br>I'm not sure at the moment, you see, because I've had a balance problem for about four years. When I was 19 they botched an operation in my ear and took all my ear out, and I lost balance completely and it took me three years to learn to walk. I did overcome all that, but since the stroke, I think the balance		**Challenges** (emphasising on the exclusion criteria, people with ear problems can be excluded if affecting their balance)

mechanism in the ear that was damaged has got worse, and then with the stroke it seemed to have made it almost not working at all. So you've got a situation where my ear, nose, and throat person said my balance now is 'Based on the right-hand ear and my eyesight.' So when you're trying to balance, because I did this balancing as well where you sit on, sorry, stand on like cushions so you don't get the benefit of the flat floor, to try and give you that effect to help you balance. Some of that did help but not very much.<br><br>**I2: Just continue doing it, P6, whatever you have you don't want to lose it. So keep doing your balance exercises, whether you make progress or not, maintain what you've got, P6. As a physio that's my viewpoint, I'm sure you will.**<br><br>Well, you must come and see me as well. Do you hear that? I said, 'You must come and see me as well.'		

**I2: All right, okay, I will. I accept your invitation, Ahmed and I we will come and see you soon.**<br><br>Okay.<br><br>**So James, if you can tell me please, how do you think the programme fits with the care from NHS? I know you have been receiving some help at some point. So how do you think our programme fits to what you have been receiving from the NHS?**<br><br>M2: I can only say it's good.<br><br>**Do you think it might [over speaking 0:51:23.8]…?**<br><br>M2: Put it this way if I come out of hospital, I had nothing, I don't know what I would be like now, because I agree with what P6 said. You've got to motivate yourself [signal breaks up 0:51:40.9].		

Can I say one thing that I think might be an advantage with this programme?<br><br>**We lost P5, P7.**<br><br>That is, if occasionally all the people involved got together physically, so that they could meet and talk and show one another how they're progressing.<br><br>**I2: That's really useful, that's how we originally planned before the pandemic, and because of the pandemic we had to start doing everything remotely.**<br><br>I know you couldn't do it then but you can start, that's coming back now that you can have groups with things like…<br><br>**I2: Yes, that's really good.**<br><br>Yes, you could meet in the park in the summer, a group of people.	**Advantages of the programme/physical meetings are suggested**	**Participants' attitude/suggestions**

**I2: Yes, good idea, yes, we should do that in the community.**<br><br>**We'll think of that for future. Sheeba, I think we just may have missed… I think we lost James.**<br><br>**I2: P5, P7, yes, I think he must have lost connection.**<br><br>**It's only a couple of questions, P6, and if James joins later we'll ask him. So would you like to continue doing the same programme on your own in the future if in now we've finished with you. So do you still want to carry out the same programme, setting goals, and try to reach them?**<br><br>Well, I do that anyway and the stroke people are still making me do it anyway. The only thing… Did I mention at the very beginning, the man I spoke to didn't answer me back. He asked me if I would like to be part of your research team,	**Desirability to continue**	

rather than be one of the people doing the research.		
**What are your thoughts about communication with us, with the research team, and was it good enough? Were you happy with our appointments and consideration of your other appointments when we book any…?**	**Happy with communication**	**Participants' attitude**
No, <u>I think in fairness, you were very, very considerate, because there's a lot of the times that I couldn't make and you went out of your way to try and compensate for me, and I appreciated that quite a lot.</u>		
**No worries.**		
So there's no question about that, you were working [over speaking 0:54:01.2].		
**It is for your benefit, we just…**		**Participants' attitude**
It was in the interest of the people you were dealing with.		

**We do that with everybody, P6. We just try to do as much as we can with you.**<br><br>No, I accept that but I know I'm not special, but you did it for me.<br><br>**I2: You are special, P6!**<br><br>You're trying to be my friend.<br><br>**I2: Hi, P5, P7!**<br><br>M2: Back, [?Jane's] got me back again.<br><br>**I2: Yes, we can't see you but we can hear you, yes.**<br><br>Now, I've just combed my hair for you.<br><br>**The question is would you like to continue doing the same programme on your own in the future? So now we have finished with you, the 12 weeks, so would you like to continue doing the same thing setting goals and try to**		

**achieve them, and encourage…?**		
**I2: Doing exercises and walking and self-monitoring, will you continue to do that, James?**	**Desirability to continue**	
M2: Yes, very much so until it becomes the norm I'll continue, and I want to do a lot of things. I've got a lad who's 15. My eldest lad is nearly 40, but I want to do. There's plenty of things. He plays football, I played football to a decent standard and I want to kick the ball around with him. I haven't done it and yes, there's lots of things that me and Jane want to do. We want to go on a Caribbean cruise, which they're all things and it all… It's just…		**Perceived benefits**
**I2: It is a really…**<br><br>It's given me another a couple of batteries in and I'm setting myself goals again, whereas before I was just going down and down and down, but now I believe that these…	**Goal setting is encouraging**	

**It's really important to have goals to motivate you. Well done, P5, P7. Well done, yes.**<br><br>M2: Very much so.<br><br>**P5, P7, the next question I think it is for you and for Jane as well. So it's about what do you think of our communication with you regarding appointments and sitting the sessions and everything. How was it?**<br><br>M2: Superb, and that's not saying that, and Jane's here now. Ahmed is just saying what was the service like?<br><br>**I2: Communication. Hi, Jane, how are you? Jane, you can come in.**<br><br>F1: Hiya.<br><br>**I2: Hello, how are you?**<br><br>F1: I'm fine, thank you, how are you?		**Perceived benefits**

**I2: I'm good, thank you. He was saying such lovely things about you in this focus group discussion.**<br><br>M2: No, I wasn't. No, oh, no.<br><br>**I2: We agreed with him because you organised everything, the whole programme involvement for him, so we were just asking P5, P7, were we communicating adequately? Were we flexible with you? How was the communication? Was it all right? Is there something that we need to work on?**<br><br>F1: <u>No, it's very good. The only thing I will say P5, P7 needed pushing several times to join in, because it was just the easy excuse not to and I nagged and nagged and he joined in</u>.<br><br>M2: I got no chance. I had no chance to escape.	**Wife pushes for exercises sessions**	**Facilitators of SM**

**I2: He did tell us he's afraid of you, so you're in trouble, P5, P7, tonight.**<br><br>F1: He does. He does need pushing a lot of the time, but once he's done it he realises anyway, it's for the best thing so.<br><br>**I2: Yes, brilliant, thank you, Jane. Thank you for supporting us. Thank you for supporting P5, P7, yes.**<br><br>F1: No worries, thank you. Thank you for including him.<br><br>**Thank you all. So the last question for everybody, how do you think we can improve this programme and move it forward a little bit?**<br><br>M2: Get two new people, joking, joking!<br><br>**I2: I was going to say that, but I can't say that, P5, P7.**<br><br>M2: You were going on about the two groups where one was		

what we were in and the other. Obviously, is that purely down to funding or whatever? I know things are difficult at the moment with things are. They're not there like they used to be there, but it would just be, as I say, the group that we were in I was so lucky and you two, you… Jane was always talking about you, and she had got it down and they would sit down and have a [makes noise 0:59:29.3], and as soon as she needed any help this way, she would just get on the phone to you. You were always there as such, you're just a phone call away. I know. I [over speaking 0:59:43.8].<br><br>**She was very good at communication; she would text us; she would email us. Yes, brilliant, thank you.**<br><br>**Yes, sometimes I reach her at work or sometimes… She's still like… She just said, 'Tell me, I'll respond to you later' and she [over speaking 0:59:58.1].**		

**I2: She's very good, yes.**<br><br>**Very supportive, yes.**<br><br>**I2: P6, is there anything you would like to change in this programme, or be added or modified?**<br><br>**P6?**<br><br>**I2: P6 is thinking.**<br><br>No, I think the only thing that I think would help is more communication in one form or another. It's one of the things I think when you say you're going to carry on doing it on your own, you always need somebody to pat you on the back and tell you that you're not doing too bad. Continuously doing it on your own ,you lose the motivation I think.<br><br>M2: In that respect, yes. I'm fortunate, I've got Jane's family. My family mainly have died.	**More communication (follow up) is suggested**	**Suggestions**

Well, I've got that many people with me, I'm training to be hermit.<br><br>M2: Jane's family, once they see me up and walking, and they're all encouraged. They all want to encourage you which is a good thing.<br><br>**I2: Yes, we can understand, P6, you can always reach out to us or to anybody in the group. Send an… We can keep in touch to see how you're doing. Maybe not push you every day, every week, but checking on you also helps, doesn't it?**<br><br>**[END OF TRANSCRIPT]**		

Focus group recording-control group1 SEPTEMBER 2022 ZS - TRIAD - 40 MINS
[MIXED RESPONDENTS]
[Other comments: The signal breaks up intermittently throughout the recording, making some of what is said unclear.]

[Moderator thanks Respondents for taking part and explains research purpose.]

If we just go around the table and everyone tell us his or her name please?

M: P10 ?.

P8, can you state you name please, again?

F1: P8 Young.

F2: [?P9 Chumba]

I2: Thank you

Transcript	Codes	Themes
**Thank you very much. So let us start with the first question. We actually want to know why did you agree to take part in this study? If you tell us about some reason or some motivations for joining this study?**<br>M: Me, yes? Just to see if I could help with other people's issues, whether the study would initially help me and help other people really. That was the initial, to see if I could try and help other people really.	**Reasons for joining the study/helping other people**	**Motivation**

**I2: Thank you P10. P8?**<br><br>F1: Sorry, what was the question, I can't hear very well?<br><br>**I2: Do you want me to phrase it?**<br><br>**Yes.**<br><br>**I2: Okay. P8, we just would like to know why did you agree to participate in this study? What was your motivation?**<br><br>F1: Well, I just thought it would help me, help other people and I've been really enjoying it. <u>It has helped me quite a bit really, because I'd had more problems and I found you very helpful.</u>	**Reasons for joining**<br><br>**Very helpful programme**	**Motivation**<br><br>**Participants' attitude**
**I2: P9, what are your thoughts? Why did you want to join the study when it was offered to you?**<br><br>F2: Like P8 and John have said, that it does help you and it will help others. <u>It encouraged you to do a bit more</u> - I found that there	**Reasons for joining**	**Motivation**

was days when I didn't do a lot of steps, but I sort of pushed myself to what extent I could, to do that little extra.<br><br>**I2: Excellent. Both of you have touched on how it helped you a little bit. P10, I'm going to go to the second question, if it was helpful for you, how was it helpful for you?**<br><br>M: I think the target setting was really good. So it enabled me to set targets. I know it sounds really daft, but at the start of it it's basic things you want to accomplish - signal breaks up 0:04:48.2]. Even down to making sure that I had the timer, so that <u>every morning it introduced me to a pattern where I knew I'd got to do something, and at night recording it.</u> <u>So that, even though it's not a lot, it helped me out.</u> So that then helped me hit different targets which changed from my initial targets, it helped me change to rather than - for an example, rather than going, I want to walk, it changed to I want to walk but I	It encouraged you to do a bit more<br><br>**Goal setting was really good**<br><br>**Helpful programme**	**Perceived benefits**<br><br>**Participants' attitude**<br><br>**Perceived benefits**

want to function. So I want to be able to do things, which then enabled me to do small targets. I think the other reassuring thing out of it was the situation where you get fatigued and you get tired and you listen to your body, and I think that was really good because a friend of mine had a stroke and he did too much and it pushed him back. So yes, you have to listen to your body. If you're tired, then you have to rest.<br><br>**I2: Thank you P10. P8, could you tell us a bit more about how this involvement helped you?**<br><br>F1: It's like P10 said, the target, I tried to do all that. Although I had quite a few setbacks, I had COVID, I couldn't do as much walking as I wanted to do, but I focused on doing what I could. Like even now, when you get tired, your body tells you what to do, you rest. Now, as I say, I am getting more stronger now and being able to do more things. So hopefully, I'll just keep carrying	**Covid affected recovery and participation in the program**	**Challenges**

on and doing the exercises, because I think exercise is really important to get your limbs going and to get focused on things.	Patient's Knowledge helps recovery	**Facilitators of SM**
**I2: Thank you, P8. What about you, P9?**		
F2: I quite enjoyed that. It's like P10 said, every morning you get up and it became like a routine. You pick up your little timer, you put it on and you think right, today - yesterday, I did so many steps, I'm going to see if I can do a little extra steps. Then there were days, like P10 and P8 says, that you had your bad days, but if I didn't do a lot of steps, what I would do was I'd put the TV on and you know the little light walking exercises, I'd try to sort of push myself slightly to do either the same amount I did or just a few more steps, up to what I could do without exerting myself. Which was good, but now, since we've taken that off, we haven't got that little motivation now, because before, we knew we had to write it down	**Self-recording of steps encourages to do more**<br><br>**Self-motivation helps to do more training**	**Perceived benefits**<br><br>**Facilitators of SM**

and how many we've done. I did cheat sometimes because I'd just stand up and stand there and I'd start going like this, doing a little walking on the step, like a little exercise. Yes, we got there. I do feel like I have gained more strength in doing the little exercises, while I was wearing that, because I'd set myself a target. If I didn't do it, I'd put the TV on and I'd just walk on the spot. Like it says, every little gets you there eventually, and we've still got to work on it.<br><br>**I2: Thank you, P9. The next question is about the little presentation that Ahmed did, right at the beginning. It was quite early on. It was just to tell you a little bit about stroke, it was to tell you about what happens after stroke and why self-management is important, and a little bit about the study. I don't know if you remember it?**<br><br>M: Yes.<br><br>F1: I'm trying to think back.	**Fear of losing motivation after finishing their participation**<br><br>**Gained more confidence to do exercises**	**Challenges**<br><br>**Perceived benefits**

**I2: P10 seems to remember it, so we'll go for P10 then. P10, what did you think about that education session? Was it easy to understand?**<br><br>M: Oh yes, it was really easy because it's mostly like target setting, when he was talking about it, in your head you could - in my head, I could see what I could possibly do or not do. So yes, it was very good. In fact, that's probably one of the main things that made me join in, to be honest, yes.<br><br>**I2: P8, did you hear my question? I'm taking you back, just after you joined the study, Ahmed did a presentation for you regarding stroke and mobility and self-management and all of that. Was that helpful for you? Was it easy to understand?**<br><br>F1: Oh yes, it was easy to understand, yes. I tried to do my best with it but, as I say, I had	**Education session was very good/a motivation for joining**	**Participants' attitude**

one or two setbacks. No, I found it very helpful.<br><br>**I2: So what about you, P9?**<br><br>F2: I'm still trying to think, I can't remember.<br><br>**It was the first time we met on Zoom.**<br><br>**I2: P8 was there as well, I think, both of you.**<br><br>F2: I think what it was, I joined in afterwards, so I might have missed. So can you repeat your questions and then I'll see what I can answer.<br><br>**I2: It's all right, if you don't remember, we don't want you to make up any answers. It's all right, don't worry.**<br><br>F2: I'm thinking now, where was I?!<br><br>**It's all right, it was three months before, so don't worry about it.**	**Education session was easy to understand**	**Participants' attitude**

**I2: How did you like or find your involvement in the goal planning session? We set goals for you and we asked you what you would like to do. So how did you find that involvement in the goal setting?**<br><br>M: For me…<br><br>F2: Oh, sorry, carry on P10.<br><br>M: No, I wasn't sure whether I was going first every time! The physio session was really good, because that made me realise what I couldn't do! Which was good because then that helped set the target. For an example, I know I wanted to walk and we went on holiday to the Isle of Aaron, and I knew I could walk, I didn't have an issue walking, it was going uphill, downhill, different areas of walking and balance really. So yes, so I did really well with that. That's down to the physio session.<br><br>**I2: What did you think about setting your own goals, P8? I know you said you moved your**	**Goal setting was really good**	**Participants' attitude**

**targets and you changed your targets, but how did you find that? Was it okay?**<br><br>F1: Yes, it was okay. I've been going to a gym as well. Been doing that. So that's helped strengthen my arms and legs. I did find the walking - I would have liked to have done more, but it was just how it happened, some days I couldn't do very well at all.<br><br>**I2: Okay, what about the goals we set together, we decided you'll be doing…?**<br><br>F1: <u>Oh yes, I did reach those. I think I've told you, I go dancing</u>. I did quite a few dances [signal breaks up 0:13:19.6]. Then I got up to - oh, I went to the shops and back a couple of times on my own and did shopping. Then of course I had the COVID and it took my confidence, but I've started now to go back again, shopping and doing - not like I did before, but I was getting there. I've been anaemic as well. So that's held me back. I've been	**Goals achieved**	**Perceived benefits**

in hospital with anaemia, but I think they're getting that under control now. So hopefully I'm going to go up now, hopefully.<br><br>**I2: Good. P9, what did you think about setting goals with us?**<br><br>F2: My goals, I think I've achieved, I'm nearly there. I do walk - me and my husband we go out for a little walk, or when we've got the grandkids, it's encouraging for them because when they're inside, you know what they are, they just want to go out. I'm driving on my own now. I feel confident in driving. The only thing is, I'm back to work now and I do get my ache, it's just my arm. I was off work for about three weeks. I've just gone back this week. Other than that, I feel quite proud that I have done my targets and it's only - the thing is trying to keep going. Even though we've met them, not 100 per cent, push ourselves, not overdo it but just take ourselves one step above there so we can achieve them and	**Covid affected recovery and participation in the programme**	**Challenges**

encourage other people to do it, but not overdo it, if that makes sense.<br><br>**I2: Yes, totally, thank you P9. So when we were setting goals and I was asking for goals, you all came up with goals straightaway, but generally when you set goals, did you find any difficulty? Was it hard for you to think of a goal for yourself?**<br><br>F2: At that time, you didn't know what your first priority was. So I just said, oh this and this. Obviously I didn't put them in all my priority list. Then slowly, you know what goal you want to achieve first, what is your main goal first for you to achieve, and then the others automatically just follow on. You do them, so you're not really over pushing yourself, because you don't want to set yourself back. When your body does tell you, you've obviously got to slow back down, then carry on with it.	**Prioritising of goals might be a challenge at goal setting**	**Challenges**

**I2: That's useful. What about you and P10, P10 and P8, when you set yourself goals, either with us or by yourself, do you find it challenging? Do you find it difficult to frame goals and say this is what I want to be doing?**<br>M: With me, I didn't go too far with them. Every day it would be, at the beginning, it would be walking down the steps. It would be going upstairs holding the rail, and then the next time it would be going upstairs without holding the rail. So little, tiny steps to begin with. Then, obviously going down the steps, but turning and, like I was saying earlier, not just walking but actually functioning and doing stuff. Trying to bend over, because initially I couldn't go anywhere out of my balance, if I went anywhere out of my base then it was awful. So I'd do little things, like one of my tasks was to re-build the trailer outside. I did that slowly, I didn't do it in one day, I thought right, I'm going to do a little bit at a time. That teaches you to do things		

without realising you're doing them, like turning, bending over, flexing, so it helped with coordination. So functioning, giving yourself somewhere to go but something to do when you get there, whether it be two minutes or an hour, I found really…<br><br>**I2: Really useful, P10. P8, did you have any problems setting the goals for yourself or with us?**<br><br>F1: What I did find, I was very nervous about going up steps, but I think I've overcome that now. I still have to hold on the rail, which I didn't do before, but I still haven't got the confidence to walk up without holding anything. No, I think I've achieved most of the goals. I've done my dancing, what I wanted to do. I've been to the gym and got up to seven with my legs and my arms, and that's helped a lot. So yes, and I'm going out now, walking, and I'm socialising more. So yes, I think I have reached the goals that I wanted		

to. I don't know whether I'll ever be like I was, but I keep trying to push myself so I can get back to how I was before.<br><br>**I2: Did you all have any help from carers while you were doing whatever you were doing to improve yourself? Did your husband help, P8? Did your partners help?**<br><br>F2: Yes, my family was quite supportive. My kids would come over. If there were things I couldn't do, they used to say, 'Mum, take your time, don't push yourself.' It's like P8 says, she walks up the stairs with the banister, I think we've got used to it. In our mind now, because we've got used to holding on to it, I even do that. I think eventually, when we just imagine if it's not there, we can do it. <u>Sometimes, if you've got somebody at the back of you, like your partner or your kids or somebody, just to give that little support, it does make a lot of difference</u>, because you look at them and you think, you know	**improvement in socialisation and participation**<br><br>**Family/carer support**	**Perceived benefits**<br><br>**Facilitators for SM**

what, I've got to do it for my kids, for my husband, for whoever is important in your life. You look at them sometimes and you think you don't want anybody pitying you.<br><br>**I2: Yes, I agree yes.**<br><br>F2: So you think, yes, that little extra helps.<br><br>**I2: What about you, P8, was Bernard around you when you were doing your activities?**<br><br>F1: Oh yes.<br><br>**I2: How did you find that?**<br><br>F1: He's been very supportive. Like the lady said. He's been behind me, making sure that I don't fall, things like that. He's very helpful. He'll do anything that I ask. No, I'm very lucky.		
**I2: Brilliant, thank you. What about you, P10, did you have any carer support?**	**Presence of carer helps safety**	**Facilitators of SM**

M: No, I get on with it myself, [unclear words 0:21:14.0].<br><br>F2: That's men for you!<br><br>F1: Good for you.<br><br>M: If I couldn't do it, I'd do little steps to make myself do it. Jill was really good with the [unclear word 0:21:30.3]. I don't know how P8 and P9 got on, but I remember that with words, talking, that I'd know what the word was but it wouldn't come out. I'd have to go and find it in my head. It might be a different word that I wanted, but it would mean the same thing. Jill would give me the time; I'd stop talking and I'd have to go and find whatever I wanted to say. She was brilliant, absolutely brilliant at letting me have the time to do that. Whereas I have found that other people, they'll try and finish your sentence for you, which is a bit annoying, and you have to go, 'I know what it is, just give me the - I will find it, but you have to give me the		

McGowan transcribe + translate

time.' That's where Jill was brilliant, yes.<br><br>**I2: Okay, good. Moving on to the next question, was there anything that you faced as a challenge, when you were recording your stuff on the paper, or when you were motivating yourself to do it, was there any challenges or any facilitators, anything that supported you to do this part of the study well? Including the pedometer and goal setting and everything that you did, was there anything that you see as a barrier or as a supporter or a facilitator, when doing these things for the study?**<br><br>M: You have to be aware of hitting the target. Say you do 2000 steps one day, you can't get into that of saying, oh I've got to do 2500 the next day, because you might feel different. You might be in a different scenario. That's where I learnt that okay, I might be able to do 1500 steps today, it might only be that it		

McGowan transcribe + translate

looks like I've done 1000 steps, but actually doing other things in those steps, i.e., a bit of gardening, a bit of tidying, then that's what I took from it, was the fact that it's now about the amount of steps, it's what you accomplish within those amount of steps.<br><br>**I2: Yes, you use it as a tool to support you.**<br><br>M: Yes, and a perfect tool, yes, really good.<br><br>**Especially that P10, I think you are one of the best participants regarding walking ability, so you are doing good with your walking.**<br><br>M: Is that me?<br><br>**Yes. So I think you have been considering other activities like gardening or maybe doing some housework, plus walking. Where other people might have their main focus on walking and number of steps.**		

M: Yes. I think that's what helped me the most, not just walking but forcing myself to function, but not getting overtired with it, actually having to move out of my base. That's where the issues were. It wasn't just walking, it was making dinner, cooking, ironing. To start with I was cooking beans, because I could do beans, I couldn't do full English dinner, because I'd be too tired cooking that many things at the same time, typical man I suppose, one job at a time.<br><br>**I2: Thank you, P10. P8, did you find anything acting as a barrier or something that helped you to do all the steps that you had to do for the study, like using the pedometer or setting your goals? Did you find anything challenging or supportive?**<br><br>F1: I can't think really. I just sort of got on with it. I thought what I've got to do, I've got to do what I can, and if I couldn't do it I		

thought well, leave it today and try another day.<br><br>M: Yes.<br><br>F1: So that's just what I did really.<br><br>**I2: Okay, very good. What about you P9?**<br><br>F2: Once I'd put it on, you'd just forget it, because the first early days was like I kept looking, after a few steps I kept looking to see what I've done. Then it's just on your mind, oh no, I haven't done a lot. So then there were days, I'd just put it on and just forget about it, do what I need to do. The only thing I thought was, you know you'd plan your day sometimes. You'd think to yourself, oh, I'll just for a walk, I'll do that. I think the only barrier was when it was raining, you didn't want to…<br><br>M: Did you say when it rained?<br><br>F2: When it was raining, yes. When it rained, you didn't want		

to go out. Then you're doing your cooking and whatever, and then you do remember - it got to days where sometimes you didn't even know you had it on. Then when you do remember, you have a look to think oh, have I don't this many? Like you say, tomorrow I'll do a bit more, or I'll do my little steps, walking on the step, to get them extra steps.<br><br>**I2: Unfortunately, we can't do much about the rain in this country, can we P10?! Sorry about that. So moving on to the next question, I hope you understand that some people got additional exercises in addition to the pedometer and the goal setting. That's how the study designers - what do you all think about it? Should we be doing that, about the design of the study?**<br><br>M: I think it's down to the individuals. Once you've done your assessment with your physio, they got me right, I think. That's important, that you actually - you're the	**Raining is a barrier for walking outside**	**Challenges**

McGowan transcribe + translate

professionals, at the end of the day. Even though each one of us have had a stroke and we're all different. So I think that's assessing to the individual, I think.<br><br>**I2: What were you going to say, P9?**<br><br>F2: I think even though we had the pedometer on, I think we should just encourage ourselves to carry on as if we have got it on and do our little extra steps if we can.<br><br>**I2: There was another group of people who had some exercises with Ahmad, that was the design of the study, but that's how the research is carried out. Any thoughts on that?**<br><br>M: I thought my physio sessions were really good. I had physio from the hospital as well, but when the physio came out and put me through all those tests, it encouraged me to try and prove that I was better than the first	**Pedometer encourages participants to do more steps**	**Facilitators of SM**

time she did it, if that makes sense?<br><br>**I2: Yes.**<br><br>M: Those exercises were really good.<br><br>**I2: Okay, in spite of not getting exercises directly from us in the study, you had exercises from the physio. What about you, P8, what do you think the design - I don't want to put words in your mouth, but what do you think about the design of some people getting extra and some people getting less, but in the same study?**<br><br>F1: I don't know. I did what I could, do you know what I mean? I would have liked to have done more, but how I was, I just couldn't. I am sort of now trying to make up for it, now that I feel better.<br><br>**I2: How does this programme that you've done with us, fit in with the NHS programmes? Does it actually resonate with**		

**the NHS programme? Is it additional? How does this fit in?**<br><br>M: For me, I think it's additional. In a positive way, I think it was really good. Again, you're thinking maybe this could help somebody else, you don't know. Yes, I thought it was excellent additional help, yes.<br><br>**I2: Thank you, P10. What about you, P8, what do you think? How did this programme fit in with your NHS care plans?**<br><br>F1: It actually fits in quite well. I'm just pleased that I'm doing it really, because it gives you so much support.<br><br>**I2: What about you, P9, how does it…?**<br><br>F2: It fitted in quite well, and the only thing I found was if we were trying to get through to the NHS, you wouldn't be able to get through them that quick. When you rang your physio, Marie, she	it was excellent additional help to what received from the NHS<br><br>**It provides so much support**	**Participants' attitude**

was there, on the other line, advising us. You guys, yourselves, if there was any problems, you said to us that we could get in touch with you. You think the exercises are little, but it could mean a great to somebody else to achieve them goals. Sometimes, you think oh, anybody can walk up and down, but it's when you actually start doing the assessment. I found, in my first assessment, I thought I could do it, but just walking up and down, where your marking was, and you were saying, turn around, I couldn't do it. So what seems little, sometimes is really very hard to achieve at that time.<br><br>M: Yes.<br><br>**I2: Obviously you would have seen the improvement after the final assessment?**<br><br>F: Oh yes. I think [?Emmett] probably knew, the first assessment he had to wait outside and I couldn't do it. They had to come back; I was just so exhausted. Which now, when I	**The programme provides quicker support and easier communication with the therapist**	**Participants' attitude**

think, it's just walking in your living room, up there, walking back, but it was too much for me. Now you could do that with your eyes closed.<br><br>**I2: Oh, excellent, that's very good. Moving on, all that we've set up for you, the goal setting, walking every day, pedometer and stuff like that, would you like to continue this, even after the study finishes?**<br><br>F2: Yes, if it will help somebody.<br><br>**I2: What about yourself?**<br><br>F2: I've got a Fitbit so I watch my steps on there.<br><br>**I2: Very good. What about you, P10, would you be continuing your goal setting?**<br><br>M: Yes, I think every day. Every day you have to goal set, with different things, yes. It might be that I take a fence panel out. You don't know. Every day, I give myself a target. It's really	**Desirability to continue**	**Perceived benefits**

important. I think that's about the [signal breaks up 0:33:20.6]. I haven't got that physical thing anymore, but I still make myself a target.<br><br>**I2: Very good. What about you, P8, would you be continuing your goal setting and counting your steps every day?**<br><br>F1: Oh yes. Yes, I would.<br><br>**I2: Brilliant. So you've learnt something and you're taking something away from it. It's going to come back on a reflection on us, I mean it's mainly on Ahmad, so Ahmad can close his ears if you want to say something bad about him! What are your thoughts about communication with the research team? Jessica came, was she all right, about booking and communication generally? What did you all think about Jessica and Ahmad and myself?**<br><br>F2: Good communication.		

F1: Yes.<br><br>F2: Supported us right through. You encouraged us. It's like I said, you was always on the other line if we had any concerns.<br><br>**I2: Brilliant, thank you. What did you think, P8?**<br><br>F1: Yes, I think very good. Got no complaints at all. You're very good. You've been very supportive and very helpful. It's nice to know, if you're needed you're at the other end of the phone. Very good.<br><br>**I2: We were reachable. What about you, P10, we were reachable, were we…?**<br><br>M: Yes, same again, but I quite liked the fact that you weren't in my face all the while. Even though I knew you were there, that you weren't hassling, you weren't going how's it going? You set the target.<br>Yes, and you just left us to it.	**Good communication**	**Participants attitude**

F1: Left us to it, yes, that was very good, yes.<br><br>**I2: Is there any way we can improve this programme that we are testing out right now? We know we have a couple of patients on this, but we would like to get your input on how could we make this better? Any thoughts?**<br><br>M: I'm thinking.<br><br>F1: I can't think, no.<br><br>F2: I think there's nothing really that you could change.<br><br>F1: No.<br><br>F2: You've been very supportive, like you said, always been there, you're on the other side and, like P10 said, you haven't been in our faces and you haven't sort of said no, this has got to be done now, within this time. You've given us that time to achieve.	**Liked online communication and follow up protocol**<br><br>**Happy with protocol (can't think of anything that need to be changed)**	**Participants' attitude**<br><br>**Participants' attitude**

F1: That's right, yes.<br><br>**I2: The whole idea was everybody wants to get better. We wanted to use your self-motivation to improve yourself, rather than us doing things for you, because the NHS is not going to work with us every day, so it's about encouraging yourselves to do it, and I think you've done that well.**<br><br>F1: At the end of the day, it's up to the individual isn't it, really?<br><br>F2: What they can handle, yes, P8.<br><br>F1: Yes, definitely. You've got to help yourself as well.<br><br>M: You knew, in a little way, you were being slightly monitored, so that encouraged you to go oh, maybe do that because I know somebody - even though you weren't there all the time, they were going to be assessed.		

**Yes, you know we will come back for you?**<br><br>M: Yes.<br><br>**I2: Just a cheeky question at the end, do you know anybody else who has participated in the study, that you've spoken to about the study?**<br><br>M; No.<br><br>F2: Well now I know where you were saying there was a couple of us in the study.<br><br>F1: No.<br><br>F2: You didn't mention no names, but I think from when we last had our Zoom meeting, I did see P8, so I knew P8 was in it, but I didn't know anybody else who was in it.<br><br>M: I think I was with another chap, so I don't know. There was just me and another chap.		

**I2: So there was obviously no chances to ask what they're doing?**<br><br>M: No. I think that's a big thing that I've learnt from having this stroke, is nobody's the same. People that haven't had a stroke assume that everybody's going to be exactly the same, and they're not.<br><br>**I2: Okay, that's lovely. That's really good to know. We ran out of questions, so I just want to say a big thank you for your time, for your help with this research, for your help with Akhmed's PhD and everything you've done for us. Akhmed will keep in touch to monitor your progress. After six months, he'll come and do an assessment to see if you're doing any better.**<br><br>**Three months from now.**<br><br>**I2: After three months.**<br><br>**Three months from now we will contact you again for last**		

**assessment, to see how you are doing on long-term, after our study.**<br><br>**I2: Just to see if you have improved from where you started in the first time, and he can show you the results and you can see what difference your self-management, your self-motivation has made, so that you can keep working even after we stop the study. So yes. So lovely to get to know you three. If there's anything else, you can get in touch, you know where we are.**<br><br>M: You'll have to let us know when he gets his PhD.<br><br>**Thank you.**<br><br>M: It would be nice to know that.<br><br>F2: Yes, definitely.<br><br>**We'll keep you posted as well. We have your details. So if any paper came out of the study, or something, we will keep you updated.**		

F2: Oh, that's good.<br><br>F1: That's good, yes.<br><br>**We are really appreciating your effort and your input in this, and we hope everyone will get the benefit. I hope you will be improving after your stroke and we also get to complete the study.**<br><br>**[END OF TRANSCRIPT]**		

