# Supplementary material for: Feasibility of a self-management intervention to improve mobility in the community after stroke (SIMS): A mixed-methods pilot study
Source: PLoS One. 2024 Aug 13;19(8):e0286611. doi: 10.1371/journal.pone.0286611 (PMC11321569; doi:10.1371/journal.pone.0286611)
Supplement: S2 File — (DOCX) [file pone.0286611.s002.docx]

## Full/long title of the study

Feasibility of a self-management intervention for improving mobility for patients following stroke in the community.

Short study title/acronym

Self-management intervention for mobility for people with stroke.

## Protocol version number and date

Version 10. Dt 28.04.2021

## Research reference numbers

| **IRAS Number** | 269079 |
| --- | --- |
| **Sponsor reference number** | ERN_18-0628 - RG_19-140 |
| **ISRCTN number** |  |
| **REC reference number** |  |

This protocol has regard for the HRA guidance

## Signature page

The undersigned confirm that the following protocol has been agreed and accepted and that the Chief Investigator agrees to adhere to the signed University of Birmingham’s Sponsorship CI declaration.

I agree to ensure that the confidential information contained in this document will not be used for any other purpose other than the evaluation or conduct of the investigation without the prior written consent of the Sponsor

I also confirm that I will make the findings of the study publicly available through publication or other dissemination tools without any unnecessary delay and that an honest accurate and transparent account of the study will be given; and that any discrepancies from the study as planned in this protocol will be explained.

Chief Investigator:

Signature: .......
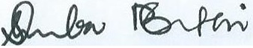
............................................................................... Date: ....24.11.2020

Name: (please print): .............SHEEBA ROSEWILLIAM............................................................................

## Sponsor statement:

Where the University of Birmingham takes on the sponsor role for protocol development oversight, the signing of the IRAS form by the sponsor will serve as confirmation of approval of this protocol.

## Table of Contents

[Full/long title of the study 1](#_Toc508371618)

[Short study title/acronym 1](#_Toc508371619)

[Protocol version number and date 1](#_Toc508371620)

[Research reference numbers 1](#_Toc508371621)

[Signature page 1](#_Toc508371622)

[Sponsor statement: 1](#_Toc508371623)

[Table of Contents 2](#_Toc508371624)

[Key study contacts 3](#_Toc508371625)

[Study summary 3](#_Toc508371626)

[Funding and support in kind 4](#_Toc508371627)

[Role of study sponsor and funder 4](#_Toc508371628)

[Roles and responsibilities of study management committees/groups and individuals 4](#_Toc508371629)

[Protocol contributors 4](#_Toc508371630)

[Study flow chart 4](#_Toc508371631)

[Study protocol 5](#_Toc508371632)

[1. Background 5](#_Toc508371633)

[2. Rationale 5](#_Toc508371634)

[3. Theoretical framework 5](#_Toc508371635)

[4. Research question/aims 5](#_Toc508371636)

[4.1. Objectives 5](#_Toc508371637)

[4.2. Outcome 5](#_Toc508371638)

[5. Study design and methods of data collection and data analysis 5](#_Toc508371639)

[6. Study setting 6](#_Toc508371640)

[7. Participant recruitment 6](#_Toc508371641)

[7.1. Eligibility Criteria 6](#_Toc508371642)

[7.1.1. Inclusion criteria 6](#_Toc508371643)

[7.1.2. Exclusion criteria 7](#_Toc508371644)

[7.2. Recruitment target 7](#_Toc508371645)

[7.2.1. Size of recruitment target 7](#_Toc508371646)

[7.2.2. Recruitment technique 7](#_Toc508371647)

[7.3. Recruitment 7](#_Toc508371648)

[7.3.1. Participant identification 7](#_Toc508371649)

[7.3.2. Consent 8](#_Toc508371650)

[8. Safety reporting 8](#_Toc508371651)

[9. Ethical and regulatory considerations 9](#_Toc508371652)

[9.1. Assessment and management of risk 9](#_Toc508371653)

[9.2. Research Ethics Committee (REC) and other Regulatory review & reports 9](#_Toc508371654)

[Regulatory Review & Compliance 9](#_Toc508371655)

[Amendments 10](#_Toc508371656)

[9.3. Peer review 10](#_Toc508371657)

[9.4. Patient & Public Involvement 10](#_Toc508371658)

[9.5. Protocol compliance 11](#_Toc508371659)

[9.6. Data protection and patient confidentiality 11](#_Toc508371660)

[9.7. Indemnity 11](#_Toc508371661)

[9.8. End of study and archiving 12](#_Toc508371662)

[9.9. Access to the final study dataset 12](#_Toc508371663)

[10. Dissemination policy 12](#_Toc508371664)

[10.1. Dissemination policy 12](#_Toc508371665)

[10.2. Authorship eligibility guidelines and any intended use of professional writers 12](#_Toc508371666)

[11. References 13](#_Toc508371667)

[12. Appendices 13](#_Toc508371668)

[12.1. Appendix 1- Required documentation 13](#_Toc508371669)

[12.2. Appendix 2 – Schedule of Procedures 13](#_Toc508371670)

[12.3. Appendix 3 – Amendment History 13](#_Toc508371671)

## Key study contacts

**Chief Investigator:** Dr. Sheeba Rosewilliam

Email: s.b.rosewilliam@bham.ac.uk , Tel: 01214158367

**Study Co-ordinator:**

**Research Team:**

1. Mr. Ahmad Sahely

Email: axs1638@student.bham.ac.uk , Tel: 01214158367

1. Dr. Andrew Soundy

Email: a.a.soundy@bham.ac.uk, Tel: 0121 414 8385

1. Mrs. Carron Sintler

Email: carron.sintler@bhamcommunity.nhs.uk, Tel: 0121 4666176

1. Ms. Caroline Hawkins

Email: caroline.hawkins@bhamcommunity.nhs.uk, Tel: 0121 466 6630

## Study summary

**Introduction:** Individuals’ lives after stroke are affected by multiple levels of disability. Appropriate rehabilitation services can help them to regain their functions and improve their quality of life. In the UK, there has been a tendency toward the early supported discharge from hospital after a stroke with the aim of providing care at home after discharge. However, stroke survivors’ needs after discharge from the hospital have been unmet as the health care services lack sufficient resources. Self-management strategies have been developed to help stroke survivors improve their self-efficacy and independence and play an active role in their rehabilitation process. The efficacy and appropriateness of self-management as an additional component to the current practice have been examined in several contexts, but there has been limited research around self-management strategies to improve walking after stroke.

**Aim:** The aim of this study is to develop and examine the feasibility of implementing a new self-management-based intervention to improve functional mobility for stroke survivors after their discharge from the hospital.

**Methods:** A self-management intervention based on a literature review and clinicians’ input has been developed with the aim to improve the mobility of people with stroke in the community. A feasibility study will follow to examine the feasibility of implementing the new intervention using a mixed-methods approach. The mixed-methods design will include feasibility randomised controlled trial and focus groups. Data will be analysed using descriptive and statistical approaches for the quantitative data and content analysis for the data from the focus groups.

## Funding and support in kind

| **FUNDER(S)**  (Names and contact details of ALL organisations providing funding and/or support in kind for this study) | **FINANCIAL AND NON FINANCIALSUPPORT GIVEN** |
| --- | --- |
| **Local resources from within School of Sports Exercise and Rehabilitation Sciences.** | **Academic support fund from School** |
|  |  |

## Role of study sponsor and funder:

University of Birmingham is the sponsor for this study.

## Roles and responsibilities of study management committees/groups and individuals

The study steering group will include the Chief investigator, Clinical co investigators, Dr. Andrew Soundy, and Mr. Brin Heliwell. The group will meet once in two months to monitor the rate of recruitment, challenges faced and implement strategies to help with recruitment.

Delivery of intervention will be monitored by the SG members throughout the study, taking into consideration any safety, protocol breeches, adverse events and duly report them to the Sponsor, REC and HRA. The SG will have an overview of the ongoing data analysis and review summary of findings every six months. SG will have an input in where and how the findings will be disseminated.

Patient & Public Involvement Group:

a) Patient-experts (BH and PEW) had a stroke several years ago. They have agreed to be a part of the PPI group for this study. They have experience of working with other research protocols. b) Thirty people who survived stroke from Northfield stroke association community club have also been consulted regarding this study and c) Doctors, physiotherapy, occupational therapy, nursing and SALT clinicians from the site where the study is recruiting from have also been consulted as stakeholders and have offered to contribute to the study design and documentation.

## Protocol contributors

The protocol was developed by the academic researchers in collaboration with the clinical therapists and the patient expert Mr.BH.

KEY WORDS:

Stroke, Self-management, Walking, Community rehabilitation, Self-efficacy, Goal-setting.

## Study Flow Chart

Informed consent gained

Randomized

# Assessments

Follow-up assessment at 6 months

Follow-up assessment at 6 months

Second assessment for control group at 3 months

.Second assessment for treatment group after intervention

# Approach and Recruitment

# Patients

# Assessment

# Intervention

Allocated to intervention

Receive self-management intervention for three months.

Allocated to control

Receive education

.Baseline assessment for treatment group

Baseline assessment for control group

Approached for study

Consent to be contacted

Screen Potential participants screened

# Enrollment

# Study protocol

Self-management intervention for improving mobility for patients following a stroke in the community: a protocol for a feasibility study

Background

Stroke is a complex health condition that results annually in death or disability of about 14 million people globally (‘Global Burden of Disease Result tool’, 2016). It occurs suddenly due to a problem in the blood supply to the brain and results in long term consequences for the survivor. In the UK, stroke is estimated to occur in more than 152,000 individuals annually (Stroke Association, 2016). It is currently estimated that there are about 1.2 Million stroke survivors living with different levels of disability subsequent to a stroke (Stroke Association, 2016). The annual cost of stroke in the UK is about £26 billion which includes health care services and the reduction in economic productivity (Patel *et al.*, 2017). The prevalence of stroke in the UK is anticipated to increase by 123 % by 2035 and that will increase the demand on the National Health Services which are already under pressure (Patel *et al.*, 2017). The increase in the incidence of stroke is attributed to the increase in the elderly population as the age is the most important risk factor for stroke (Stroke Association, 2016 p: 10). Moreover, the increase in awareness about the early symptoms of stroke and the improvement in medical interventions that help save patients’ lives at stroke onset contribute to increased survival rate following a stroke (Bray *et al.*, 2013; Heart and Stroke Foundation, 2017).

Following a stroke, about half of the people who survive need help with their daily activities as a result of various impairments that affect their physical, cognitive, emotional, and social well-being (Feigin *et al.*, 2008; Heart and Stroke Foundation, 2017). A major physical disability involves limitations in an individual’s lower limb activities such as balance and mobility (Heart and Stroke Foundation, 2017; West and Bernhardt, 2012). Mobility has been found to be a key problem for two-thirds of people after stroke and can lead to depression and isolation (Lawrence *et al.*, 2001; Pinter and Brainin, 2012). In a systematic review of qualitative studies that discussed the lived experience of stroke survivors with physical rehabilitation, most of the participants have considered mobility as the most important goal of rehabilitation after stroke (Luker *et al.*, 2015). This reflects the importance of being mobile for people with stroke to enable their independence after discharge.

Rehabilitation is deemed to be essential for the recovery process after stroke as it helps to improve the impaired functions resulting in a better quality of life for stroke survivors (Veerbeek *et al.*, 2014). There has been an array of rehabilitation paradigms ranging from applying biomedical models to considering patients’ psychosocial status in planning for their rehabilitation (Cameron, 2010). However, there is increasing evidence that rehabilitation based on a bio-psychosocial model of care can result in better outcomes for the patient due to the holistic yet individualised approach to the management of the condition (NHS England, 2016). The ideal care plans based on this model are pertained to cover the health condition of an individual with consideration to her/his personal and environmental factors (WHO, 2001). Yet, in stroke rehabilitation, it might be challenging for therapists to provide ideal plans that cover individual needs of the survivors especially at the time of discharge from acute care where they need more of emotional and social support (Pearce *et al.*, 2015).

The studies that discussed survivors’ experiences after stroke demonstrate that survivors need support as they might feel lost and neglected by the NHS after discharge from stroke units (Pearce *et al.*, 2015). The National Clinical Guideline for Stroke Care has described the point of transfer from hospital to home as very stressful for people with stroke and their family/carers (ISWP, 2016). Previous studies that discussed stroke survivors’ experience with rehabilitation highlighted several barriers for their recovery such as lack of enough resources, feeling left out, confused, and unable to manage their daily lives while waiting for the rehabilitation services (Stroke Association, 2015). The Early Supported Discharge (ESD) services can contribute to reducing this abandonment felt by patients after discharge (Fearon, Langhorne and Trialists, 2012).

Particularly, the ESD system has been implemented to reduce the time of patient’s stay at the hospital and to provide their rehabilitation at home with the same intensity of care as if they are inpatient at the hospital (SSNAP, 2017). The ESD has been known for its cost-effectiveness and promoted by healthcare policy in the UK and other developed countries for people with long-term conditions such as stroke (SSNAP, 2017). Studies that evaluated the ESD’s impact on patients have reported positive outcomes of increasing their self-autonomy, meeting their needs, and feeling more in control to carry out their own routines at home instead of being inpatient and having a passive role within healthcare (Fearon, Langhorne and Trialists, 2012). However, it is clearly evident that further training can improve the rate of recovery and outcome especially during the first six months after stroke (Lohse, Lang and Boyd, 2014).

Increasing patients’ capability of self-management can work in parallel with the ESD to better address patients’ needs and facilitate their integration into a new life after stroke (Pearce *et al.*, 2015). Self-management (SM) has been recommended as an approach that empowers the role of patients in facilitating their recovery in addition to the usual care provided by professionals and leads to optimal rehabilitation outcomes (Warner *et al.*, 2015). In the last decade, various self-management interventions have increasingly shown a positive effect on survivors’ self-efficacy, emotional and functional recovery, and social participation (Warner *et al.*, 2015; de Silva, 2011). SM has also been shown to play a role in reducing the length of stay in hospitals (Lorig *et al.*, 1999). Self-management has been defined as ‘‘a process in which individuals acquire skills, strategies and knowledge to manage the physical, psychological, emotional and social effects of a chronic condition’’ (Sadler *et al.*, 2017 p:1). Emma *et al* (2014), based on their focus groups that examined the role of SM for patients with stroke, argued that SM is an implicit part of life post-stroke and does not need a new label. But, they identified some factors that can facilitate or hinder patient’s ability for SM such as an individual’s capacity, support for SM, and SM environment.

SM is mediated by self-efficacy which reflects individuals’ beliefs and confidence in their own capability to perform a particular behaviour (Zimmerman, Bandura and Martinez-Pons, 1992). Self-efficacy has shown a high correlation with mobility, activities of daily living, and quality of life in stroke population and has been negatively associated with depression (Korpershoek, van der Bijl and Hafsteinsdóttir, 2011). Hence, based on these self-efficacy principles SM has shown a positive impact on the physical, psychological, and social rehabilitation outcomes (Warner *et al.*, 2015). Although SM has been recommended by the national stroke guidelines to be included in the rehabilitation plans, it has been identified that SM interventions are not adequately implemented in current practice (Saunders *et al.*, 2016). Pearce *et al* (2015) did a systematic review of the qualitative studies that discussed the experiences of stroke survivors with SM support following stroke and they indicated that future qualitative research should focus on informing SM support interventions and its implementation because SM interventions have the potentials to address various emotional and psychological needs of survivors throughout different stages of recovery.

Previously, interventions have applied SM principles to influence patients’ skills, knowledge, and practice and ranged from simple strategies for providing patients with information about their conditions to more complex interventions aimed at behaviour change (Parke *et al.*, 2015). The interventions that target behaviour change led to more effective outcomes (de Silva, 2011). A systematic review done by Warner *et al*. (2015) to explore the effectiveness of SM interventions to improve the functional activities and participation for stroke survivors found goal setting and follow-up, providing individualised structured information and professional support as the most effective interventions. The effectiveness of SM interventions might be varying based on the patient’s rehabilitation context. Therefore, the development of a SM intervention should be patient-centred and suitable for the patient’s individual context (Clark et al., 2018).

SM interventions for improving the mobility of stroke survivors in the community are uncommon (Lennon, McKenna and Jones, 2013). Recently, there have been some studies that used SM, some of which incorporated interventions to improve mobility outcomes after stroke. Examples of these interventions include the Rehabilitation Training (ReTrain) intervention (Dean *et al.*, 2018), the Extended Rehabilitation Service (EXTRAS) for **s**troke patients (Shaw *et al.*, 2015), the Independent Mobility-related Physical Activity (IMPACT) Program (DePaul *et al.*, 2014), and the Bridges self-management programme (McKenna *et al.*, 2015). The results of the previous studies confirmed that SM interventions delivered for individuals or in groups in the community can be effective for people after stroke. However, there is still no comprehensive SM mobility intervention that can be easily embedded within the current practice of stroke rehabilitation. Not all of the previous interventions were directed toward improving functional mobility outcomes alone. Rather, some of the studies used generic programmes to shape the rehabilitation plan in addition to the usually provided care and relying on the therapists’ role for an extensive number of therapeutic sessions and follow up. For instance, the ReTrain programme included about 25 contact sessions each of which needed therapists supervision (Dean *et al.*, 2018). The IMPACT had less time of supervision in the community as it aimed at starting the SM interventions for stroke survivors while they are inpatients, but the interventions do not continue for an adequate time after discharge where the patients need more support (DePaul *et al.*, 2014). Also, patients need to visit a rehabilitation hospital or an outpatient facility for follow up and assessment. The EXTRAS was developed based on the SM principles to provide long term support for stroke survivors without focusing on mobility as the key goal of rehabilitation (Shaw *et al.*, 2015). The Bridges study had a limitation in the recruitment plan and was criticised for not having an adequate number of participants for examining the effectiveness of the SM components. Building on the previous work in this area of research, this study is aimed at reducing the extent to which the busy therapists at public services are required to contact patients. This will particularly avoid what the ReTrain study has applied in term of employing exercise trainers for extensive periods of time (Dean *et al.*, 2018). From another perspective, examining effectiveness of SM interventions has been an area of discussion as some of the previous studies used a small sample size on which it might be difficult to draw a conclusion (Warner *et al.*, 2015). The proposed study will include an adequate sample size to test the feasibility of the new intervention based on what has been suggested in the literature and based on the study context.

Rationale:

Considering the increasing prevalence of stroke among the elderly population in the UK and the rising incidence of stroke in younger patients (Stroke Association, 2018), there is a need for more effective rehabilitation delivery systems incorporating increased self-management strategies. However, reduced resources in the current healthcare climate causes challenges in addressing patients’ needs for long term care (Stroke Association, 2016; Teasell *et al.*, 2012). It has been estimated that 45% of stroke survivors feel neglected in the community and are in need of support when they are discharged from stroke care facilities (Stroke Association, 2016). Self-management interventions have the potential to help in addressing this gap. Although they have been recommended by NICE guidelines, self-management interventions for improving community mobility are uncommon (Lennon, McKenna and Jones, 2013). This study tries to address this gap in current practice by developing a self-management intervention for mobility in the community and to test its feasibility in the local practice to improve stroke survivors’ independence in their functional mobility.

**Aims and Objectives: *The primary aim*** of this study is to develop, implement, and test feasibility of a self-management intervention to improve functional mobility for stroke survivors in the community.

Theoretical framework

Self-management strategies have been developed to help stroke survivors improve their self-efficacy and independence and play an active role in their rehabilitation process. The efficacy and appropriateness of self-management as an additional component to the current practice have been examined in several contexts, but there has been limited research around self-management strategies to improve walking after stroke.

Research question/aims

1. To what extent it can be feasible to implement a self-management intervention to improve mobility of stroke survivors in the local context of Birmingham?
   1. Objectives

The objectives of this study are:

1) To implement the intervention in community stroke rehabilitation settings following which the feasibility of the new intervention will be evaluated using a mixed-methods study design. The specific feasibility objectives are to:

1. explore participants’ perspectives about the acceptability, practicality, and fidelity of the new intervention
2. scope out methodological feasibility of a future randomised control trial
   1. Outcome

A number of functional, emotional, and social outcomes will be measured followed by focus groups with participants at the end of the study to explore their lived experiences with the proposed intervention. The assessments will be carried out for all participants by an independent blinded assessor at the community centres or in their homes if they are unable to attend.

a. Feasibility outcomes

Feasibility outcomes will focus on examining the fidelity, acceptability, and practicality of delivering the new intervention. This will be measured using some feasibility related outcomes such as:

- Estimating the recruitment and retention rates
- Examining randomisation and blinding
- Evaluating the eligibility criteria
- Measuring participants’ adherence to the intervention and follow up
- Testing the feasibility of the self-management and performance outcome measures

b. Efficacy outcomes

***The primary outcomes*** will include outcomes that are related to the constructs of self-management such as self-efficacy, goal-achievement, the ability for self-monitoring and evaluation, and providing feedback. Self-efficacy will be measured by using the stroke self-efficacy questionnaire (Jones, Partridge and Reid, 2008). The Goal Attainment Scale (GAS) will be used to measure participants ability to achieve their goals (Hurn, Kneebone and Cropley, 2006). Level of adherence and performing of other self-management related elements will be examined using the Patient Specific Functional Scale, the recording diaries of physical activities, and action planning documents.

***The secondary outcomes*** will include outcomes that can determine the impact of the new intervention on participants’ level of functional, emotional, and social performance and other mediators. In particular, the Functional Gait Assessment (FGA), 6-metre walking test, maximum walking speed, distance walked using pedometer will be used to track the changes in functional mobility. These outcome measures have been tested reliability and validity to detect changes in stroke population (Lam *et al.*, 2010; Lin *et al.*, 2010). To examine the emotional well-being, the Montreal Cognitive Assessment and General Health Questionnaire-12 will be used (Hardy *et al.*, 1999; Cumming *et al.*, 2013)

c. Qualitative Evaluation

This will include focus group interviews with the study participants to evaluate the fidelity and acceptability of the new intervention. Focus groups will use an interview guide that will include questions to explore acceptability, practicality, sustainability, effectiveness, and ways to improve intervention from participants’ perspectives. The participants in the study will be assigned to one of four focus groups and each group will include 6 to 9 participants.

Study design and methods of data collection and data analysis

***Study design***

The objectives of the study will be addressed by incorporating different research methodologies throughout different stages. A scoping review of the available literature regarding self-management strategies to improve mobility was carried out at the design stage of the study. This provided the foundational knowledge to build the mobility SM programme based on evidence. The clinical therapists in the local NHS trust and patient experts were consulted regarding the components for SM for mobility training. Following this development of the SM programme, a mixed-methods feasibility study using the sequential explanatory design (Creswell 2014) will be carried out to examine the feasibility and acceptability of the new intervention. This feasibility study is planned so that researchers can test the intervention and methodology to refine or modify the intervention and the research methodology for large-scale studies (Thabane *et al.*, 2010).

The use of mixed-methods designs is considerable in research methodology because of their capacity to provide a deeper understanding of the research problem and strengthen the study’s conclusion (Onwuegbuzie and Leech, 2006). While quantitative methods can be used to generate numerical data from experimental or observational studies, the inclusion of qualitative methods in the same study helps in understanding and exploring of participants’ lived experiences, behaviours, perceptions, and thoughts about an intervention, problem, or phenomenon (Harding and Gantley, 1998). In this study, mixed-methods will include a prospective feasibility Randomized Controlled Trial (RCT) and focus groups after the delivery of the intervention.

The feasibility RCT will be single blinded (assessors blinded) and will include two arms, one for the treatment group where the newly developed SM intervention will be applied and the other for the control group in which the participants will receive the usual care from the NHS. The participants will be randomly allocated to treatment or control groups by an administrator using computer-generated randomisation chart.

Following the feasibility RCT the focus-groups will be done to explore participants’ perspectives on the effectiveness of the new intervention, their experience and adherence to intervention protocol, identify factors affecting its feasibility and implementation for future large scale RCTs and to contribute to refinement of the intervention. The selection of focus groups instead of individual interviews for qualitative data collection followed the suggestion of Powell and Single (1996) who believe that the focus groups are better when the objective of a study is to understand participants’ responses in relation to social context or interaction with other individuals with shared experiences, such as a community based intervention. The advantage of using focus groups lies in the stimulation of participants’ thoughts and reminding them of feelings and perceptions about the same topic through the dynamic interaction between them (Stewart and Shamdasani, 2014).

***Data collection***

The treatment group will receive the self-management intervention for mobility training for three months in addition to the usual care. The control group will receive the usual care provided by the local NHS and community services. Both groups will receive education about walking safely using a power point presentation.

Data collection will involve physical assessments including Functional Gait assessment, Timed up and Go, 10mts walk test, patient specific functional scale, 6 minute walking test and Pedometer recordings. Psycho-social testing will include MOCA, Stroke self-efficacy scale, GHQ-12 and Goal-attainment scaling. The research team members will carry out the assessments at the beginning of the study, at three months and at six months. These objective assessments have been tested for reliability and validity and for the purpose of this study have been integrated into a single document attached. Demographic data such as participants’ name, date of birth, gender, diagnosis and GP address will be collected at baseline. Self-reported data on the goal-setting documents, recording diary and usual care record will be completed by the participant and collected by the researcher. Assessments will be carried out at the beginning of the study, at three months and at six months. Data will be collected on paper copies and transferred to electronic files at University by the research team members. The paper copies will be stored in the researcher’s office at the University of Birmingham and only the research team members will have access to the records. Due to the COVID pandemic the data will be collected on electronic copies of the forms using emails or using zoom and telephone questioning. These are the safety considerations for doing the assessments over zoom and phone. Only participants at a moderate level of disability are included in the study (the person is at least able to walk with a maximum of one person assistance, with or without a gait aid). For the physical assessments (Functional Gait assessment, Timed up and Go, 10mts walk test, 6 minute walking test) if they are not able to assume the starting position or meet the basic requirements such walk without someone, they will be stopped from doing the test. During testing, carers will be asked to stay with participants. The questionnaires (patient specific functional scale, MOCA, Stroke self-efficacy scale, GHQ-12 and Goal-attainment scaling) will be done online or completed using interviews.

The study data will be collected over a 24-month period and data analysis will extend to another year. As and when a treatment group of 10-12 participants is formed they will start intervention. Four or five such treatment groups will be set up. Similarly, as and when a control group of 10-12 participants is formed they will be assessed and have the education session as a part of the study. Four or five of these intervention groups will be set up.

Participants from both the treatment and control groups will be invited for focus group discussions which will be held at the community center or university facilities. Due to COVID pandemic the focus groups will be conducted on online meeting applications such as zoom. Only those who consent will be given a date and time for the group discussion. There will be two focus groups (each with 6-9 people) consisting of participants from treatment group and two focus groups with control group participants. The focus group guide will be used to collect data that will be recorded using a digital recorder that is encrypted. In case of zoom focus groups, the recording will be done using the facility available on zoom. Both audio and video will be recorded as zoom provides encryption for audio and cloud recordings. Having video will facilitate people to talk to each other as if they are in a meeting room. Zoom recordings are safe because the recordings are encrypted, password protected and access to these files on the cloud is restricted. These recordings will be downloaded and saved on to University servers for extra security. In line with the originally approved protocol, the zoom recordings will be transferred to university computers within three days and deleted from the cloud. The recordings on the University server will be kept till they are transcribed and then the audio and video files will be deleted. The anonymous transcriptions will be stored for 10 years according to the University guidelines.

The recording will be transferred to university computers and the audio files deleted from the digital recorder within 3 days. The recordings will be kept on University servers till they are transcribed. The audio files will then be deleted. The anonymised transcripts will be kept for 10 years till the data is analysed and written up.

Participants will be given instructions on how to join a zoom meeting in the participant information sheet. They will also be guided to set up zoom on their phone or laptop over the phone. The researcher will trial zoom with them before the actual meetings take place.

***Treatment Group***

The intervention will be delivered for the treatment group in community centres by the researcher on specific days of the week over a period of 12-weeks. Due to the Covid pandemic this intervention will be delivered using zoom. A qualified/registered therapist will be on site to support the researcher. To improve safety for zoom classes a qualified researcher and registered therapist will be delivering the classes together. The assessments will show us the level at which each participant can perform the exercises and hence the exercises will be customised to the participants’ ability. For e.g. if an exercise requires a person to stand, if the person is weak then he/she will be asked to do the exercise in sitting. The exercise booklet is also designed to customise exercise for different levels of ability of participants. Considering safety, the size of the groups has been further reduced to 5-8 in each group so that we can monitor them better. Participants’ carers will be invited to accompany patients for additional support, safety and to encourage self-management strategies at home. Additionally, exercising safely at home leaflet will be given to them and explained to them. Environmental modifications such as standing next to a furniture or wall will be considered for slightly weaker participants. Participants will be reassured that the risks of doing exercises remotely over zoom are lower compared to independent home exercises given by NHS as they are being remotely monitored on zoom and following risk assessment additional safety measures mentioned above have been put in place.

Participants can use community transport or their own transport to travel to the community centres. Since participants are recruited from a single NHS trust which covers a specific geographical area, the distance they travel for the study intervention will be similar to the distance they travel for NHS physiotherapy.The developed SM intervention will include education sessions on improving mobility, self-management components including goal-setting, action-planning, peer support, home exercises booklet, self-monitoring using recording diary, and five group exercise sessions for mobility described below. The participant will complete a recording diary and goal setting record that will be provided to them as paper copies. Due to the COVID pandemic all the paperwork will be collected on electronic copies of the forms using emails or using zoom and telephone questioning.

The schedule for the activities for the treatment group is set out below. The individual components of the study intervention are described following this.

**Week 1:** (Group and Individual interventions)

• Education about improving gait, self-management and safety will be done using a power point presentation at the community centre for participants in treatment groups. Due to the COVID pandemic the education sessions will be delivered through zoom.

• Self-management strategies including individualised goal-setting for mobility goals and action-planning.

• Patient will be taught home exercises using a booklet and self-monitoring of mobility training using a recording diary; both documents have been developed from published work.

**Weeks 2****, 4, 6, 8 &10: (Group)**

The researcher will conduct one group session once every two weeks in weeks 2, 4, 6, 8 and 10 for 10-12 people where participants are taught exercises for strengthening, balance, and mobility. Due to the COVID pandemic this number will be reduced to 5-8 people in each group. The exercises will be based on standardised exercise booklets.

Three patient-experts will provide peer-support during the group sessions. The PI and the NHS trust’s Patient Advice and Liaison services will train patient-experts on the protocol, and positive reinforcement strategies using a standard format. The patient-experts are stroke-survivors.

**Weeks 3, 5, 7, 9 &11: (Individual)**

Phone call by the researcher to review goals and progress.

**Week 12: (Group)**

Reiteration of safety, reviewing mobility-training and positive reinforcement.

1. **Patient education**

For successful implementation of self-management programmes, there is a need for increasing patients’ knowledge about their own health condition, understanding the importance of any specific activities that they can take control of, and having the required skills to perform these activities (Warner *et al.*, 2015). At the beginning of the intervention, participants will be engaged in an education session on how to improve their mobility based on their functional level after stroke. The sessions will focus initially on educating and providing patients with the required information regarding the benefits of task-specific practice on their walking recovery, safety considerations, strategies for strength, balance and endurance, and how to deal with different challenges during the recovery period. The participants will also receive information about the appropriate management of physical symptoms that are related to stroke such as fatigue, weakness, and pain. Due to the COVID pandemic the education sessions will be delivered through zoom.

1. **Self-management components**

**Goal Setting**

Goal setting is known as a process in which stroke survivors and their carers are engaged with the stroke rehabilitation team to identify individual treatment goals that are meaningful, challenging and valuable to the survivors (Rudd *et al.*, 2016). In this study, an individualised goal-setting of mobility related goals will be targeted for each participant based on their baseline assessment. The participant will fill in the goal-setting document on their own or with the help of the family member. The goals on this document will be reviewed every two weeks by the participant along with the researcher. The researcher will try to highlight goals that are realistic and easy to obtain in short and long term. Their goals will be recorded, reviewed and periodically updated if needed. Due to the COVID pandemic the goal-setting documents will be completed electronically using emails or using zoom and telephone questioning.

**Action Planning**

After goal setting, participants will negotiate appropriate action plans for their training with the researcher. These action plans will be filled in the goal-setting and action planning document by the researcher. The action plans will be based on motor control principles of physical training after stroke to be repetitive, challenging, and task-specific (Kleim and Jones, 2008). Selection of activities for an action plan will be done collaboratively by the researcher and the participant and will be related to the identified goals, consider participant’s safety and functional capacity, and focus on walking-related activities. Participants will have the choice to set a specific time and place for their training at home and will be provided with a home exercise booklet as a guideline for their training. Due to the COVID pandemic the action-planning documents will be completed electronically using emails or using zoom and telephone questioning.

**Group-exercise sessions**

The intervention will include five group-exercise sessions. The exercise sessions will run once every two weeks. Due to the Covid pandemic this intervention will be delivered using zoom. This element of the intervention will be based on evidence and integrates suggestions from therapists and patient experts reached out by the research team as a part of Patient and Public Involvement (PPI) (Clark *et al.*, 2018). The group sessions will focus on exercises for strengthening, balance, and mobility along with peer discussions. Each group will include at least 10-12 participants and will be in addition to the individualised home exercise plan for each participant. Due to the COVID pandemic this number will be reduced to 5-8 people in each group. It is possible that the participants will not be able to attend all the group exercise sessions. Registers will be kept for the group sessions to find out the adherence to group exercises.

**Self-monitoring and periodical review of goals**

Patient engagement and motivation are primary objectives for the newly developed intervention and will be embodied by facilitating participants’ independence and taking an active role in their recovery process. Based on motor learning theories, it is recommended that people who engage more in the learning process can behave and learn better especially when they get a chance to evaluate their own performance, receive feedback, and get the opportunity to correct their own errors (Schmidt, 1982). Participants will be taught to self-monitor their completion of planned activity training. Using a recording diary, participants will document their daily performance and evaluate their progress in relation to the previously identified goals. The recording diary includes variables such as type of exercise, compliance per week, level of confidence and issues faced in their daily training. The participants will be reassured in the participant information sheet that they can do the exercises to the maximum ability possible, but it is not compulsory. Since this is a feasibility study for self-management we will be able to estimate adherence to home exercises using the self-monitoring record.

**Biweekly phone calls**

Participants will be asked to rate their ability to perform the planned activities using the Patient Specific Functional Scale (Stratford, 2014) in bi-weekly phone calls with the researcher to encourage and guide their progression. The role of the researcher in this step is to generally review the participant’s progress and to encourage any small success when a goal is achieved to enhance the motivation and self-efficacy of participants.

1. ***Control Group***

The control group will receive the routinely provided care in the community services for stroke survivors after their discharge from the stroke care unit. The usual care in this context is guided by the regulations of the NHS and is anticipated to follow the national clinical guidelines for stroke care. The participants of this group will receive an education session about walking safely at the beginning of the study (week1) using a power point presentation. Due to the COVID pandemic the education sessions will be delivered through zoom. The participants will be asked to describe what they have received from the NHS as a usual care for their rehabilitation at baseline and at three months later and at six months. Due to the COVID pandemic all the paperwork will be collected on electronic copies of the forms using emails or using zoom and telephone questioning.

***Data analysis***

As the study is anticipated to use a mixed methods design, there is a need for using quantitative and qualitative approaches to analyse the collected data. A descriptive statistical analysis will be used to describe the result of the feasibility and performance-related outcomes (Lancaster, Dodd and Williamson, 2004; Pagano and Gauvreau, 2000). For instance, frequency counts such as mean, standard deviation, median, and minimum-maximum values will be used to summarize the statistics of feasibility testing. Data can be presented through different forms of tables, bars, or graphs. Inferential statistics with test for differences will be used to compare the data of participants within and between the two groups of the study. Specific tests such as Wilcoxon rank sum or Spearman correlation might be used in case of non-parametric or if the data is not normally distributed. Multiple way ANOVA might be used to compare between all participants in regard to any outcome or mediating factor. Intention to treat analysis will be considered to prevent the effect of drop out. It is important to note that the aim of the data analysis for the efficacy outcome measures is to evaluate the feasibility of using these outcome measures and the role of context (how, by whom, and when they can be measured), keeping in mind that feasibility trials are underpowered to detect clinically important treatment effects (Arnold *et al.*, 2009). For the qualitative data from focus group interviews, a qualitative content analysis approach will be used to present the identified themes from participants discussion about the new intervention (Graneheim and Lundman, 2004).

Study setting:

The intervention will be delivered in community settings for the treatment group patients in the study.

Participant recruitment:

- 1. Eligibility Criteria

The participants will be stroke survivors who are discharged from acute hospitals in the West Midlands (UK) to continue their rehabilitation in their homes or under community services. To maximize the chance of recruitment, the target population for the study will be recruited from different stroke community rehabilitation teams. They will be purposefully selected using a criterion-based sampling as they need to have a certain level of mobility and cognitive ability to participate in this study.

In addition to recruitment from the community hospital above, we will recruit people with stroke in the community. This will be done to improve our recruitment to the study as patients being admitted for stroke in the NHS trust have reduced due to the pandemic situation.

Inclusion criteria

Individuals will be eligible to participate in the study if they:

- Are adults (18 years to 99 years).
- Have had diagnosis of a first stroke.
- Are within the first six months post-discharge from the hospital
- With Functional Ambulation Category (FAC) ≥ 2. This means that the person is at least able to walk with a maximum of one person assistance, with or without a gait aid (Mehrholz *et al.*, 2007).
- Have cognitive capacity that allows them to communicate and consent to participate in the study. The Montreal Cognition assessment (MoCA) (Cumming *et al.*, 2013) and the FAC will be used for screening. Moreover, clinicians will be involved in determining patient’s ability to participate prior to approaching any participant to this study.
- Can understand English. However, feasibility of including participants who speak Punjabi or Urdu will be explored using translated information sheets and interpreters when possible using family members who speak English.
- Having access to a suitable device that can launch ZOOM Programme.

Exclusion criteria

Patients will be excluded if they:

- Are unable to participate or unable to give consent to participate in the programme due to marked cognitive impairment.
- Suffer from severe cardiopulmonary diseases or severe arthritis or have other major co-morbidities, which might be a risk for community walking.
- Have severe aphasia.
- Have severe spasticity or fatigue post stroke.
  1. Recruitment target

Size of recruitment target

As no formal sample size calculations are required for feasibility studies (Thabane *et al.*, 2010) and the objectives of pilot trials focused on feasibility can often be met with relatively few numbers of participants, the study will include a sample of 45 participants for each group of the study with a total of 90 persons with stroke. In similar studies, it has been confirmed that sample size estimates should not be derived based on the estimated effect of interventions on a clinically important outcome, instead, sample size estimates should be based upon the specific objectives and outcomes of the pilot trial (Arnold *et al.*, 2009). For instance, the sample size estimation for this study is based on current statistics of patients discharged over a year and allowing for some level of attrition and withdrawal from the study. Moreover, it has been recommended for pilot studies to have at least 30 data sets to be able to estimate any variations in the outcome variables of the study (Browne, 1995). The ideal sample size for the focus group has been varying in the literature based on the contextual variables of a study such as the nature of topic, background of the participants, and the level of facilitator expertise (Doody, Slevin and Taggart, 2013). However, the suggested range for focus groups in nursing research has been ranged between four and twelve participants. This study will select between six and nine participants for each of the four focus-groups.

Recruitment technique

Criterion-based sampling will be used since participants need to have a certain level of mobility to participate. The sample size is based on current statistics of patients discharged over a year, i.e. 216 (1/3 ineligible and 75% consent rate) with 108 potential participants. For the focus groups those participants who are willing to come for the group discussion will be invited.

- 1. Recruitment

Participant identification:

Stroke survivors will be identified by the clinical therapists who are members of the patients’ clinical care team, from their referral lists or patient registers and screened for eligibility using the inclusion criteria. The therapists in the settings will be inducted to the study and criteria familiarised before the study begins. Some of the inclusion criteria will be available to the therapists from the patients’ case notes and others through talking to the patient. The therapists are qualified to assess the inclusion criteria as these are available routinely in stroke patients’ notes. The Clinicians informed that MOCA cognitive assessment is routinely being done in the hospital settings. All forms of treatment will require capacity to consent and hence these are also routinely recorded in patients’ notes.

Then potential participants will be approached by their therapists at the Moseley Hall hospital which will act as Patient Identification centre. The clinical therapists will give study leaflets and gain consent from patients to be contacted by the researcher. If patients agree to be contacted, then the potential participant’s name and contact details will be passed on to the researcher.

In order to improve our recruitment to the study we will additional recruitment strategies listed below:

1. Place request and study information for interested participants on the websites of the Stroke association and Different Strokes.
2. Stroke association volunteers to give study leaflets to people they meet in the community.
3. Advertise in local magazines/newsletters in Birmingham,
4. Place leaflets and posters in identified GP surgeries and
5. Request GPs to hand out study leaflets to patients with stroke.

Contact details of the researcher will be available on the leaflets, posters, study information left in these areas. These documents will also list the inclusion criteria. Interested persons can contact the researcher directly.

Consent:

Informed consent will be obtained by the Researcher after giving them study information sheets in person. The researchers who are taking consent have undergone the good clinical practice training given by the NIHR for research in 2019. The study information sheets given by the researcher at this point will have information on risks, benefits and reassurance that they are not obliged to participate, and their care will not be affected if they do not participate. Potential participants would have been checked by clinicians for mental capacity to understand and retain information for them to be approached for participation in the study. Potential participants will be given two days to consider the information. They will be given the researcher’s contact details to contact for clarifications about the study information prior to being approached for consent. Then they will be given the consent forms to sign and if they are unable to sign due to arm impairments their carer can sign in the presence of the participant and the researcher. Consent forms will be signed either at the point of discharge in the community hospital or at patient’s home if patients prefer it. Patients will be contacted before-hand to decide where and when they want to meet to sign the consent form. Once patients sign the consent forms then participants will be assigned to one of the study groups.

Following the additional strategies for recruitment if any patient contacts the researcher directly from the community through phone or email, then the researcher will give them basic study information and screen them based on inclusion criteria over phone or zoom. If anyone is found eligible, then the researcher will send them the participant information sheet as described below.

During the COVID pandemic, the researcher will contact the potential participant over phone or online meeting application zoom to give details of study. The information will be shared by email, over phone and posted to the patients' home if they ask for it as hard copy. Instead of signing paper consent forms in person, consent forms will be signed electronically, or verbal consent will be recorded. We will have multiple options for consenting depending on the participants’ ability. We will read out the consent forms over zoom or on the phone and ask the participants for consent for each statement. We will record this in a document. According to the HRA website a witnessed verbal consent can be recorded in the documents. For the intervention study we will send the consent forms electronically and participants can either sign it electronically or send an email stating they are consenting to participate. For the focus group study, we will gain verbal consent over zoom in separate recordings. We will also keep the verbally recorded consents in an audio format in a password protected file on the University server for 10 years. We considered sending a self-addressed envelope for paper copy of the consent form but due to restrictions in mobility and pandemic restrictions we would like to avoid unnecessary trips outdoors.

Participants will also be informed that if they want to withdraw from the study, they are free to do so at any point in the study without giving any reasons. Their anonymised data will be included in the analysis, but no further data will be collected.

Safety reporting:

The previous studies provided a proof of concept for the implementation of self-management interventions for stroke survivors. The included interventions have been shown to be safe and not leading to any harmful adverse events. In our context, if any adverse events occur during the study, the participant will be told to stop, and the adverse event will be reported to the ethics board and described in the results section.

At times the researcher will have to go to patient’s homes for physical and psychosocial assessments if the patient prefers this rather than coming to the community center. The therapist will call at times suitable for the patient and ensure the office is informed of where they always go and through electronic diary sharing and keeping mobiles on. The researchers are DBS cleared for working with older adults with a check against the barred list. Participants will be asked to have chaperones if they require during assessments. Researchers will adhere to the University of Birmingham’s lone working policy if they have to assess the patient alone in their homes. During the COVID pandemic researcher will not arrange to go to the participants’ homes.

Ethical and regulatory considerations

Design and Methods: This is a feasibility study using mixed methods including randomised control design and focus groups. The study will evaluate feasibility of implementing and outcome of intervention using quantitative measures and collect subjective opinions of participants involved using qualitative method.

The randomised controlled trial is planned where participants will be allocated to two groups, intervention group and control group. Randomisation to two groups will help test the feasibility of carrying out a large scale RCT in the future. Randomisation is also important to study cause-effect relationship of intervention and outcome. Allocating participants randomly to two groups by a staff member who does not know their baseline status (blinded) will reduce chances of people with better potential being allocated to the treatment group or people with weaker potential being allocated to control group. Since the study is about self-management within group therapy, the focus groups will provide a collective understanding from the participants about the intervention. Focus groups will take place in a community center or the University of Birmingham facilities and will be conducted by an independent researcher from the University of Birmingham who will not be delivering intervention to avoid researcher bias and improve validity of results. Due to COVID pandemic the focus groups will be conducted on online meeting applications such as zoom.

Benefits:

1. The exercises have also been set up with safety considerations with chairs or other support close to the person doing the exercise. The participants are also provided safety advice in the education session and the exercise booklet.
2. Discussing goals with people will give opportunity to problem solve and be motivated to work on their goals. They will get better guidance from therapists after discharge compared to current practice where patients wait for help from NHS. Participants will be empowered to problem-solve for future situations through this exercise. With two patient experts supporting the groups, participants will be motivated and receive additional encouragement.
3. Assessments will be done by researcher to collect data regarding their physical and psychosocial well-being. These assessments can take place in the participants’ homes if they prefer. This might be seen as intrusion. However, participants’ consent will be obtained before deciding where they want to be assessed. During the COVID pandemic the assessments using outcome measures will also be done over zoom. This will also reduce their travel to the community center. The other self-recordings such as exercise record and goal-setting and action planning document will be done by the patients at their own convenience and they can take the help of their family members if required.
4. In the real world, patients are offered limited opportunities for ongoing rehabilitation and treatment in the community which causes people to feel abandoned and unable to reach their maximum potential. This situation can be improved, and the patients helped, by offering them training and opportunities to enable them to help themselves through self-management.
5. The educational element will create awareness of problems that cause difficulty in walking after a stroke and teach them the importance of exercise for improved walking for people in both the control and treatment groups.
6. The assessments will inform them of their current status with regard to walking and then they can work to improve their status. The repeat assessments at 3 months and 6 months can be motivational for them to work harder to get better.
7. The exercise classes for the intervention group will have the potential to improve the strength, balance and endurance required for better walking in the community. This will also be reinforced by the home exercise booklets they will be given to take home and practice. During the COVID pandemic the study documents will be sent over email. If the participant prefers the study documents will be posted to the participant’s home address.
8. They will benefit from participation in this study by learning to manage the chronic symptoms like spasticity and fatigue during the study and for their future.
9. The goal-setting and action planning meetings and self-monitoring using diaries for the intervention group participants will help them to problem solve and self-motivate themselves to plan their rehabilitation and take ownership of their rehabilitation. They will gain confidence to take ownership of other aspects of their health if they learn this self-management strategy.
10. Working in groups gives them a forum to discuss their challenges and provide peer support, reduces loneliness and isolation in the community.

Compliance with legislation: The researchers are trained in Good Clinical Practice for research and have training in Data protection and GDPR processes. Hence they will collect and handle data according to these guidelines.

- 1. Assessment and management of risk

Participants will have difficulties in walking due to their stroke. Hence there might be a risk of fear of falling in the group sessions. There will no be actual risk of falls as the groups are supervised by a therapist with help of family members and peer supporters. Moreover, we considered this risk and decided to have smaller groups with 10-12 people in each group whereas normally group exercises will have about 15 participants. Due to the COVID pandemic this number will be reduced to 5-8 people in each group. In order to minimise any risks due to doing exercises over zoom, an additional information leaflet with on exercising online will be given (Copyright: www.safeexerciseathome.org.au Creative Commons Attribution-Non Commercial-No Derivatives 4.0 International License). Environmental modifications such as standing next to a furniture or wall will be considered for slightly weaker participants. Exercise will be customised at lower levels depending on the participants’ ability determined by the assessments. For home exercises participants will be prescribed exercises at a level suitable to their ability, considering the level of support available at home to ensure that they do not risk falling. A risk assessment is not being carried out at each participant’s home for home exercise because home exercises are prescribed routinely as a part of NHS out-patient care and does not involve risk assessment of homes.

Despite precautions if there is a fall during group exercise sessions this will be reported as adverse event. Participant will be asked if they want to continue the session and in the study. Exercise intensity or level of challenge will be reduced if patient decides to continue. Additionally, we will also invite carers or family to provide motivational support to the patients both at the group sessions and at home. If the participant lives alone they will still be encouraged to do exercises that are a level suitable for them with safety precautions such as doing exercises next to furniture or a counter. The inclusion criteria specifies patients of certain level of ability so severely disabled patients would have already been excluded. Patient carers and family are only an additional support and they are not compulsory.

The researcher at the sessions will be assisted by a qualified therapist. Both these people are trained to watch out for risks and avoid risks.

Goal-setting will be done individually with each participant. Discussing personal circumstances to set their goals might cause a bit of distress due to reflecting on their condition. Participants will be reassured and supported if they are temporarily upset. However, if issues are raised that are not within the realm of this research, we will redirect them to our clinical partners and refer them to appropriate sources of help.

People with stroke often have pain due to spasticity and fatigue. These complications were highlighted by the patient experts involved as a part of the PPI. The participants in this study might also experience these symptoms. Following physical training these symptoms may be more severe. However, the participants will be educated on these symptoms and how to manage these symptoms.

Coming to sessions in a community centre might be effortful. However, there is limited therapy offered in the community and this study offers intervention that could benefit them. Hence, they will be encouraged to come regularly to the classes. Yet, they will not be coerced. Despite the inconvenience caused by travel to the group sessions, patient experts recommended that we have five of these sessions since group therapy has several benefits such as socialisation and peer support. Due to the COVID pandemic this exercise intervention will be delivered using zoom which will reduce the inconvenience due to travel.

- 1. Research Ethics Committee (REC) and other Regulatory review & reports

This study proposal and ethics will be reviewed by the NHS research ethics committee through IRAS. Once approved the data collection will start and follow the ethical guidelines set out. The participants’ GP will be informed about their participation in this study. The annual reports for the study will be produced by the Chief investigator for the ethics committee and CI will inform the ethics committee about the completion of the study. The annual reports will also be completed and submitted by the chief investigator to the ethics committee.

### Amendments

If there is a need for substantial amendments, the steering group will be consulted, and amendments will be submitted for approval to the research ethics committee by the CI before being implemented. The protocol and relevant documents will be updated with revised up to date version numbers. These changes will be communicated to the NHS trust’s R&D, study sponsor and participating clinical collaborators.

- 1. Peer review

The protocol has been reviewed by a senior academic within the school, reviewers from funding body (funding was unsuccessful), academics within the team and the post-graduate research committee.

- 1. Patient & Public Involvement

Two meetings were held with patient-experts (BH and PEW) on 08/04/2019 and 26/04/2019, b) Group discussions took place with 30 people in Northfield stroke association community club on 02/05/2019, and c) Presentation and discussion with doctors, physiotherapy, occupational therapy, nursing and SALT clinicians took place on 08/05/2019.

The above meetings helped refine the intervention, study design, methodological aspects of recruitment and assessment.

The community stroke-club members could not recollect any explicit self-management strategy and they had limited experience of early supported discharge. They felt this study intervention would be a good step forward. They queried whether people with more severe walking deficits could be included and whether we needed to consider the cognitive ability of patients to participate in self-management. The clinicians in their stakeholder meeting also raised the above queries. The changes made in the protocol based on these two meetings are:

• To include slightly more disabled participants (FAC 2) and to invite carers or family to involve in the self-management intervention to improve adherence and safety.

• The research therapist will discuss potential participants’ cognitive ability with the clinicians at the point of recruitment. Montreal Cognitive Assessment will be administered to assess cognitive ability.

• Clinicians also suggested an objective measure of walking such as using a pedometer, which will be given to participants. Due to COVID pandemic the pedometer will be posted to the participant’s home address.

• Due to the extent of the geographical area, clinicians suggested having another community centre for the group sessions, which is currently being negotiated.

We made the following changes based on patient-experts’ views:

• To have five group sessions instead of the originally planned two sessions since they highlighted that we often undervalue the use of group sessions.

• To have information sheets in Punjabi and Urdu due to the diversity in the geographical location of this study.

• To add the General Health Questionnaire-12 to assess psychological well-being in participants since Mr. BH stated that we tend to ignore these aspects following discharge.

• To carry out focus-groups instead of interviews for this study since participants will support each other if we collect data in focus groups and might be reluctant to speak openly in individual interviews.

• Exercises around balance, strength, and endurance along with positive feedback on their performance will be added.

• To deliver education on safe-walking for the control group participants.

• To record routine NHS therapy.

During the PPI and stakeholder meetings, we requested participants for their contribution and involvement with this work.

Mr. BH has kindly agreed to serve on the research team. He has offered to attend panel meetings with the PI, talk to potential participants to recruit them for the study, serve as a peer-supporter during the group sessions and moderator during focus group meetings. He has also agreed to help with the paperwork by reviewing documents, participant information sheet, and the lay summary.

Mr. PEW has also kindly offered to attend group sessions to provide peer support, encouragement to participants, and check documents for this study including the lay summary.

- 1. Protocol compliance

All the researchers involved in the study will be aware of the protocol and ethical aspects of the study.

Adherence to the protocol will be monitored by the chief investigator in the monthly meetings. If there are minor accidental breaches (e.g. failing to date documents) they will be corrected for the continuation of the study. If major breaches are identified they will be reported to the sponsor and guidance sought from the steering group to decide on plan of action.

- 1. Data protection and patient confidentiality

The study principal investigator (PI) and the other researcher will be responsible for the process of data collection and will ensure that the participants’ anonymised data and information will be saved on a database held securely at the School of Sport, Exercise, and Rehabilitation Sciences in the University of Birmingham, UK. The researcher will contact the participants to arrange for their meetings at different points during the study including education sessions, assessments, and focus groups.

- 1. Indemnity

Insurance and indemnity to meet potential legal liability for harm to participants arising from management of research will be arranged by the University of Birmingham who is the sponsor for this study.

Since the research is not taking place in the NHS there will be no legal liability of investigators arising due to conduct of this research. The NHS staff will not be delivering any part of the intervention or doing assessments for this study. The NHS sites are only patient identification sites for this community-based research. The NHS staff will only be giving out study leaflets and taking consent to be contacted for this study.

- 1. End of study and archiving

It is proposed that the study will end in two years in March 2022.

The hard copies of the consent forms will be stored in the academic’s office in the University. All other data will be electronic and stored in the University’s server in password protected files for 10 years according to University guidelines.

- 1. Access to the final study dataset

Only the researchers involved in the study will have access to the anonymised data sets. Permission is being sought from the participants to use their data for secondary analysis during the consenting process.

The clinical collaborators will have periodical summary of the findings in the steering group meetings.

The anonymous data might have to be deposited in public repositories as per the open access guidelines for some journals and decision will be made by the steering group committee regarding this.

- 1. Dissemination policy

The findings of this study will be disseminated through different avenues. This will include publications in related journals, conference presentations, stroke assemblies, social media, institutional reports, summary to participants and relevant charity websites. The dissemination of the study finding is anticipated to confirm whether the RCT is still an appropriate design for the future study that can compare the efficacy of the new intervention to the usual care provided by the NHS to improve walking recovery in the community.

- 1. Authorship eligibility guidelines and any intended use of professional writers

All the academics, clinical collaborators and service user (Mr.BH) will be named on the papers written following this study as they have all contributed to the design of the study, will contribute to the data collection, analysis and reviewing of the manuscript. Ahmad Sahely will draft the manuscripts along with the other research team members.

References

Arnold, D. M. *et al.* (2009) ‘The design and interpretation of pilot trials in clinical research in critical care’, *Critical Care Medicine*, 37(Supplement), pp. S69–S74. doi: 10.1097/CCM.0b013e3181920e33.

Bray, B. D. *et al.* (2013) ‘Associations between the organisation of stroke services, process of care, and mortality in England: prospective cohort study’, *Bmj*. British Medical Journal Publishing Group, 346, p. f2827.

Browne, R. H. (1995) ‘On the use of a pilot sample for sample size determination’, *Statistics in medicine*. Wiley Online Library, 14(17), pp. 1933–1940.

Cameron, I. D. (2010) ‘Models of rehabilitation commonalities of interventions that work and of those that do not’, *Disability and Rehabilitation*, 32(12), pp. 1051–1058. doi: 10.3109/09638281003672377.

Clark, E. *et al.* (2018) ‘One size does not fit all–stroke survivor’s views on group self-management interventions’, *Disability and rehabilitation*. Taylor & Francis, 40(5), pp. 569–576.

Cumming, T. B. *et al.* (2013) ‘Montreal Cognitive Assessment and Mini–Mental State Examination are both valid cognitive tools in stroke’, *Acta Neurologica Scandinavica*. Wiley Online Library, 128(2), pp. 122–129.

Dean, S. G. *et al.* (2018) ‘Community-based rehabilitation training after stroke: results of a pilot randomised controlled trial (ReTrain) investigating acceptability and feasibility’, *BMJ open*. British Medical Journal Publishing Group, 8(2), p. e018409.

DePaul, V. *et al.* (2014) ‘Independent Mobility and Physical ACtivity Training (IMPACT) on a Stroke Rehabilitation Unit: A Pilot Study’, *Archives of Physical Medicine and Rehabilitation*. Elsevier Ltd, p. e31. doi: 10.1016/j.apmr.2014.07.081.

Doody, O., Slevin, E. and Taggart, L. (2013) ‘Focus group interviews in nursing research: part 1’, *British Journal of Nursing*. MA Healthcare London, 22(1), pp. 16–19.

England, N. H. S. (2016) ‘Commissioning guidance for rehabilitation’, *London: NHS England*.

Fearon, P., Langhorne, P. and Trialists, E. S. D. (2012) ‘Services for reducing duration of hospital care for acute stroke patients’, *Cochrane Database of Systematic Reviews*. John Wiley & Sons, Ltd, (9).

Feigin, V. L. *et al.* (2008) ‘Long-term neuropsychological and functional outcomes in stroke survivors: current evidence and perspectives for new research’, *International Journal of Stroke*. SAGE Publications Sage UK: London, England, 3(1), pp. 33–40.

‘Global Burden of Disease Result tool’ (2016). Institute for Metrics and Health evaluation. Available at: http://ghdx.healthdata.org/gbd-results-tool. .

Graneheim, U. H. and Lundman, B. (2004) ‘Qualitative content analysis in nursing research: concepts, procedures and measures to achieve trustworthiness’, *Nurse education today*. Elsevier, 24(2), pp. 105–112.

Harding, G. and Gantley, M. (1998) ‘Qualitative methods: beyond the cookbook.’, *Family Practice*, 15(1), pp. 76–79.

Hardy, G. E. *et al.* (1999) ‘Validation of the General Health Questionnaire-12: Using a sample of employees from England’s health care services.’, *Psychological Assessment*. American Psychological Association, 11(2), p. 159.

Heart and Stroke Foundation (2017) *Different Strokes: Stroke recovery across the ages*.

Hurn, J., Kneebone, I. and Cropley, M. (2006) ‘Goal setting as an outcome measure: a systematic review’, *Clinical rehabilitation*. Sage Publications Sage CA: Thousand Oaks, CA, 20(9), pp. 756–772.

Jones, F., Partridge, C. and Reid, F. (2008) ‘The Stroke Self‐Efficacy Questionnaire: measuring individual confidence in functional performance after stroke’, *Journal of clinical nursing*. Wiley Online Library, 17(7b), pp. 244–252.

Kleim, J. A. and Jones, T. A. (2008) ‘Principles of Experience-Dependent Neural Plasticity: Implications for Rehabilitation After Brain Damage’, *Journal of Speech Language and Hearing Research*, 51(1), p. S225. doi: 10.1044/1092-4388(2008/018).

Korpershoek, C., van der Bijl, J. and Hafsteinsdóttir, T. B. (2011) ‘Self‐efficacy and its influence on recovery of patients with stroke: a systematic review’, *Journal of Advanced Nursing*. Wiley Online Library, 67(9), pp. 1876–1894.

Lam, H. S. P. *et al.* (2010) ‘The validity and reliability of a 6-metre timed walk for the functional assessment of patients with stroke’, *Physiotherapy theory and practice*. Taylor & Francis, 26(4), pp. 251–255.

Lancaster, G. A., Dodd, S. and Williamson, P. R. (2004) ‘Design and analysis of pilot studies:recommendations for good practice’, *Journal of Evaluation in Clinical Practice*, pp. 307–312. doi: 10.1111/j..2002.384.doc.x.

Lawrence, E. S. *et al.* (2001) ‘Estimates of the prevalence of acute stroke impairments and disability in a multiethnic population’, *Stroke*. Am Heart Assoc, 32(6), pp. 1279–1284.

Lennon, S., McKenna, S. and Jones, F. (2013) ‘Self-management programmes for people post stroke: a systematic review’, *Clinical Rehabilitation*. Sage Publications Sage UK: London, England, 27(10), pp. 867–878.

Lin, J.-H. *et al.* (2010) ‘Psychometric comparisons of 3 functional ambulation measures for patients with stroke’, *Stroke*. Am Heart Assoc, 41(9), pp. 2021–2025.

Lohse, K. R., Lang, C. E. and Boyd, L. A. (2014) ‘Is more better? Using metadata to explore dose–response relationships in stroke rehabilitation’, *Stroke*. Am Heart Assoc, 45(7), pp. 2053–2058.

Lorig, K. R. *et al.* (1999) ‘Evidence suggesting that a chronic disease self-management program can improve health status while reducing hospitalization: a randomized trial’, *Medical care*. JSTOR, pp. 5–14.

Luker, J. *et al.* (2015) ‘Stroke Survivors’ Experiences of Physical Rehabilitation: A Systematic Review of Qualitative Studies’, *Archives of Physical Medicine and Rehabilitation*. Elsevier Ltd, 96(9), pp. 1698-1708e10. doi: 10.1016/j.apmr.2015.03.017.

McKenna, S. *et al.* (2015) ‘Bridges self-management program for people with stroke in the community: a feasibility randomized controlled trial’, *International Journal of Stroke*. SAGE Publications Sage UK: London, England, 10(5), pp. 697–704.

Mehrholz, J. *et al.* (2007) ‘Predictive Validity and Responsiveness of the Functional Ambulation Category in Hemiparetic Patients After Stroke’, *Archives of Physical Medicine and Rehabilitation*, 88(10), pp. 1314–1319. doi: 10.1016/j.apmr.2007.06.764.

Meinert, C. L. (1999) ‘The CONSORT Statement’, 117(15), pp. 677–680.

Onwuegbuzie, A. J. and Leech, N. L. (2006) ‘Linking research questions to mixed methods data analysis procedures 1’, *The qualitative report*, 11(3), pp. 474–498.

Organization, W. H. (2001) *International classification of functioning, disability and health: ICF*. Geneva: World Health Organization.

Pagano, M. and Gauvreau, K. (2000) *Principles of Biostatistics*.

Parke, H. L. *et al.* (2015) ‘Self-management support interventions for stroke survivors: a systematic meta-review’, *PLoS One*. Public Library of Science, 10(7), p. e0131448.

Patel, A. *et al.* (2017) ‘Executive summary part 2: societal costs of stroke in the next 20 years and potential returns from increased spending on research’, *Stroke Association, October*.

Pearce, G. *et al.* (2015) ‘Experiences of self-management support following a stroke: a meta-review of qualitative systematic reviews’, *PloS one*. Public Library of Science, 10(12), p. e0141803.

Physicians, R. C. of (2017) ‘Rising to the Challenge: The Fourth SSNAP Annual Report’, in. Royal College of Physicians London.

Pinter, M. M. and Brainin, M. (2012) ‘Rehabilitation after stroke in older people’, *Maturitas*. Elsevier, 71(2), pp. 104–108.

Rudd, T. *et al.* (2016) ‘National clinical guideline for stroke Prepared by the Intercollegia the Stroke Working Party. 2016’.

Sadler, E. *et al.* (2017) ‘Exploring stroke survivors’ and physiotherapists’ views of self-management after stroke: a qualitative study in the UK’, *BMJ open*. British Medical Journal Publishing Group, 7(3), p. e011631.

Saunders, D. H. *et al.* (2016) ‘Physical fitness training for stroke patients’, *Cochrane Database of systematic reviews*. John Wiley & Sons, Ltd, (3).

Schmidt, R. A. (1982) *Motor control and learning : a behavioral emphasis*.

Shaw, L. *et al.* (2015) ‘A trial to evaluate an extended rehabilitation service for stroke patients (EXTRAS)’, in *SRR Summer meeting 2015*. Newcastle University.

de Silva, D. (2011) *Helping people help themselves: A review of the evidence considering whether it is worthwhile to support self-management*. Health Foundation.

Stewart, D. W. and Shamdasani, P. N. (2014) *Focus groups: Theory and practice*. Sage publications.

Stratford, P. W. (2014) *Assessing_the_patient-specific.PDF*.

Stroke Association (2016) *State of the Nation Stroke statistics*.

Teasell, R. *et al.* (2012) ‘Time to rethink long-term rehabilitation management of stroke patients’, *Topics in stroke rehabilitation*. Taylor & Francis, 19(6), pp. 457–462.

Thabane, L. *et al.* (2010) ‘A tutorial on pilot studies: the what, why and how’, *BMC Medical Research Methodology*, 10(1), p. 1. doi: 10.1186/1471-2288-10-1.

Veerbeek, J. M. *et al.* (2014) ‘What is the evidence for physical therapy poststroke? A systematic review and meta-analysis.’, *PloS one*, 9(2), p. e87987. doi: 10.1371/journal.pone.0087987.

Warner, G. *et al.* (2015) ‘A systematic review of the effectiveness of stroke self-management programs for improving function and participation outcomes: self-management programs for stroke survivors’, *Disability and Rehabilitation*, 37(23), pp. 2141–2163. doi: 10.3109/09638288.2014.996674.

West, T. and Bernhardt, J. (2012) ‘Physical activity in hospitalised stroke patients’, *Stroke Research and Treatment*, 2012(October 2010). doi: 10.1155/2012/813765.

Zimmerman, B. J., Bandura, A. and Martinez-Pons, M. (1992) ‘Self-motivation for academic attainment: The role of self-efficacy beliefs and personal goal setting’, *American educational research journal*. Sage Publications, 29(3), pp. 663–676.

Appendix 1- Required documentation

CVs of the research team

Patient Information Sheet (PIS)

- 1. Appendix 2 – Schedule of Procedures
  2. Appendix 3 – Amendment History

Amendment No. Protocol version no. Date issued Author(s) of changes Details of changes made

List details of all protocol amendments here whenever a new version of the protocol is produced.

Protocol amendments must be submitted to the Sponsor for approval prior to submission to the REC.
