# Supplementary material for: Feasibility of a self-management intervention to improve mobility in the community after stroke (SIMS): A mixed-methods pilot study
Source: PLoS One. 2024 Aug 13;19(8):e0286611. doi: 10.1371/journal.pone.0286611 (PMC11321569; doi:10.1371/journal.pone.0286611)
Supplement: Attachment — Submitted filename: Response to Reviewers.docx [file pone.0286611.s004.docx]

**Comment to the reviewer:**

Thank you very much for your thoughtful comments on the submitted manuscript. Here is the response to the comments in details:

| **Reviewer’s Comments** | **Response** |
| --- | --- |
| 1. discussion: authors need to add a short note about causes of high rate of drop off and non adherence among patients for follow up so, the number of participants had dropped in the end of the study. | Thank you for the helpful comment. The reasons for dropping off and non adherence to the follow up were added to paragraph 4 in the discussion. |
| 2. Limitations: please add relative small number of patients to the limitations of the study. | This limitation has been recognised and added to the limitations section (last paragraph). |
