# Supplementary material for: Feasibility of a self-management intervention to improve mobility in the community after stroke (SIMS): A mixed-methods pilot study
Source: PLoS One. 2024 Aug 13;19(8):e0286611. doi: 10.1371/journal.pone.0286611 (PMC11321569; doi:10.1371/journal.pone.0286611)
Supplement: Attachment — Submitted filename: Response to Reviewers.docx [file pone.0286611.s005.docx]

**Comment to the editor:**

Thank you very much for your comment on the revised manuscript. Here is the response to the comment in details:

| **Editor’s Comment** | **Response** |
| --- | --- |
| Please ensure that you refer to Figure 2 in your text. | Figure 2 has been referred two in the text (page 24 in the manuscript) |
