# Supplementary material for: Feasibility of a self-management intervention to improve mobility in the community after stroke (SIMS): A mixed-methods pilot study
Source: PLoS One. 2024 Aug 13;19(8):e0286611. doi: 10.1371/journal.pone.0286611 (PMC11321569; doi:10.1371/journal.pone.0286611)
Supplement: Attachment — Submitted filename: Response to Reviewers.docx [file pone.0286611.s006.docx]

**Comment to the editor:**

Thank you very much for your comment on the revised manuscript. Here is the response to the reviewer’s comments in details:

| **Reviewer’s Comments** | **Response** |
| --- | --- |
| Major revisions: 1- The abstract is not clearly written. The trial design is not clearly communicated. The abstract should mention that it was a two-phase sequential design with a randomized controlled study component.  2- Write the “Data analysis” section more clearly. Consider replacing the following statement. “The frequency counts such as means, standard deviations or ranges of values were used to summarise the analysed data.” with “Categorical data was summarized using frequencies and percentages. Continuous data was summarized using means, standard deviations or ranges.”  3- Data analysis section states, “The aim of the data analysis for the efficacy outcome measures was to evaluate the feasibility of using these outcome measures, keeping in mind that feasibility trials are underpowered to detect clinically significant treatment effects [28].” Identify and explain the statistical methods used to evaluate the feasibility of the study. Indicate if attaining a successful proportion of patients defined feasibility.  4- Patient characteristics section: Provide the standard deviation for age. In addition to the number of male participants, provide the corresponding percentage. Throughout the paragraph, in table 5.2 and throughout the document, provide percentages that correspond to the frequencies and provide standard deviations associated with means.  Minor revisions: 1- Page 17: Replace “said yes” with “agreed”.  2- Indicate if adverse events were collected according to a standardized method. In addition to frequencies, provide corresponding percentages.  3- The standard statistical term for average is mean. | Thank you for this comment. The design is now clearly stated in the abstract (as suggested).  The suggested replacement of sentences has been done.  An explanation of methods used to evaluate the feasibility of this study was added (with a reference to guidance paper) into this section and discussed in the results sections under the recruitment rate, retention rates and participants’ adherence to the intervention.  The standard deviation for age and percentage of male participants were added in this section. The suggested addition of percentages and standard deviations has been followed all through the manuscript.  Done, thank you.  Adverse events were collected throughout the follow up calls (every 2 weeks) and in the recording diary of each participant. This information has been clarified in the methods under the outcomes section.  It has been corrected in the “walking improvement subtheme” in the results section and checked all over the manuscript. |
| 4- To assist in the review process, add line numbering to the document.  Reviewer #4: (No Response) | Line numbering has been added, thank you. |
