# Supplementary material for: Feasibility of a self-management intervention to improve mobility in the community after stroke (SIMS): A mixed-methods pilot study
Source: PLoS One. 2024 Aug 13;19(8):e0286611. doi: 10.1371/journal.pone.0286611 (PMC11321569; doi:10.1371/journal.pone.0286611)
Supplement: Attachment — Submitted filename: Response to Reviewers.docx [file pone.0286611.s007.docx]

**Comment to the editor:**

Thank you very much for your comment on the revised manuscript. Here is the response to the reviewer’s comment:

| **Reviewer’s Comments** | **Response** |
| --- | --- |
| Reviewer #4: The authors demonstrated enough knowledge and understanding about the subject matter. However, adding the p and eta square values to the tables would have been greatly appreciated | Thank you very much for reviewing our manuscript again and for your constructive comments. We thought we should explain that we feel this comment might not be applicable to the objectives of our manuscript.  The comment suggested that we include p and eta squared values to the tables; however, we clearly stated in the method and discussion sections that this study was not powered enough to estimate any clinical effect of the intervention as a feasibility study. Although the manuscript has demonstrated some trends toward improvement in participants’ functional and psychosocial outcomes, the results cannot offer any conclusion about the effect of the intervention. A sentence was added to the data analysis section to clarify this point.  Please note that our methodological decision was informed by the following literature that guide the development and conduct of feasibility studies.   - According to Professor Sim, between group effects from pilot studies ‘will be imprecise’ and ‘do not allow a robust decision on a main trial’.   Sim, J. Should treatment effects be estimated in pilot and feasibility studies?. *Pilot Feasibility Stud* **5**, 107 (2019). <https://doi.org/10.1186/s40814-019-0493-7>.   - The aim of our study was to scope out design and protocol implementation as evidenced by this review by Arnold, D.M. *et al.* (2009) ‘The design and interpretation of pilot trials in clinical research in critical care’, *Critical Care Medicine*, 37(Supplement), pp. S69–S74. Available at: <https://doi.org/10.1097/CCM.0b013e3181920e33>.     Thank you again for your effort on our improving our manuscript. |
